# Impact of CNS Diseases on Drug Delivery to Brain Extracellular and Intracellular Target Sites in Human: A “WHAT-IF” Simulation Study

**DOI:** 10.3390/pharmaceutics13010095

**Published:** 2021-01-13

**Authors:** Mohammed A. A. Saleh, Elizabeth C. M. de Lange

**Affiliations:** Division of Systems Biomedicine and Pharmacology, Leiden Academic Center for Drug Research, Leiden University, 2333 CC Leiden, The Netherlands; m.a.a.e.w.saleh@lacdr.leidenuniv.nl

**Keywords:** blood–brain barrier, passive transport, CNS diseases, brain pharmacokinetics

## Abstract

The blood–brain barrier (BBB) is equipped with unique physical and functional processes that control central nervous system (CNS) drug transport and the resulting concentration–time profiles (PK). In CNS diseases, the altered BBB and CNS pathophysiology may affect the CNS PK at the drug target sites in the brain extracellular fluid (brain_ECF_) and intracellular fluid (brain_ICF_) that may result in changes in CNS drug effects. Here, we used our human CNS physiologically-based PK model (LeiCNS-PK3.0) to investigate the impact of altered cerebral blood flow (CBF), tight junction paracellular pore radius (para_radius_), brain_ECF_ volume, and pH of brain_ECF_ (pH_ECF_) and of brain_ICF_ (pH_ICF_) on brain_ECF_ and brain_ICF_ PK for 46 small drugs with distinct physicochemical properties. LeiCNS-PK3.0 simulations showed a drug-dependent effect of the pathophysiological changes on the rate and extent of BBB transport and on brain_ECF_ and brain_ICF_ PK. Altered para_radius_, pH_ECF_, and pH_ICF_ affected both the rate and extent of BBB drug transport, whereas changes in CBF and brain_ECF_ volume modestly affected the rate of BBB drug transport. While the focus is often on BBB paracellular and active transport processes, this study indicates that also changes in pH should be considered for their important implications on brain_ECF_ and brain_ICF_ target site PK.

## 1. Introduction

Both the rate and extent of central nervous system (CNS) unbound drug transport determine CNS concentration–time profiles of the unbound drug (PK) [1]. PK at the CNS target sites in the brain extracellular fluid (brain_ECF_) and brain intracellular fluid (brain_ICF_) is a function of plasma PK, drug transport across the blood–brain barrier (BBB), and intra-brain distribution. Such PK processes result from the combination of the drug physicochemical properties and the physiological characteristics of the CNS [2,3].

The BBB lies at the brain microvessels, including brain capillaries and their direct surroundings [2]. The BBB has physical properties that reduce passive drug transport across the BBB for hydrophilic and large molecules, i.e., by the presence of the tight junctions between the brain microvascular endothelial cells. In addition, pericytes and astrocyte end feet ensure a complete coverage of the brain microvascular endothelial cells, while the basement membrane surrounds the endothelial cells and pericytes, separating them from each other and from the astrocytes end feet. All together, these cells ensure the physical integrity of the BBB against the foreign plasma molecules. The BBB also has active efflux and influx transporters, pinocytosis, transcytosis, and metabolic enzymes, which are all powered with energy supplied by the large mitochondrial count. The brain tissue composition and active cellular membrane transporters further determine the unbound drug PK in the different brain compartments, while different pH values of the CNS compartments govern, for acids and bases, the extent of ionization [2].

CNS disease pathophysiology may result in altered (unbound) brain PK, as has been shown, for example, for traumatic brain injury [4,5], epilepsy [6], and brain tumors [7]. The brain_ECF_ and brain_ICF_ unbound PK govern the CNS drug effects; therefore, understanding the impact of pathophysiological changes associated with CNS diseases on brain PK target sites is indispensable.

While being a very important parameter, Kp_uu,BBB_ is a measure for the extent (at equilibrium) but not the rate of drug transport. However, for drug effects, also the profile of concentrations seems of importance [8], i.e., having the right concentration, for the right duration, at the right site. Therefore, CNS drug development should consider the effect of both the rate and extent of BBB drug transport and of intra-brain distribution processes on the target site PK, and as indicated above, these processes may be influenced in CNS disease conditions.

Physiologically-based pharmacokinetic (PBPK) modeling [9] has provided important insights in what governs PK at different CNS sites in health [10] and in disease [11]. PBPK models use a system of ordinary differential equations to predict the rate of change of drug concentration in each physiological compartment. Importantly, PBPK models are mechanistic and explicitly distinguish between physiological body compartment characteristics (such as tissue volume, blood flow, etc.) and drug properties (such as molecular weight, lipophilicity, pk_a_, etc.). Body organs and tissues are mathematically represented as compartments with their physiological volumes, and these are connected to the central blood circulation by their physiological blood flows. Physiological processes involved in drug transport and disposition such as active transport, metabolism, tissue non-specific binding, etc. are mechanistically included. Given their mechanistic nature, PBPK models allow the translation between species and between populations and the exploration of different virtual scenarios, i.e., what-if scenarios.

**Figure 1:**
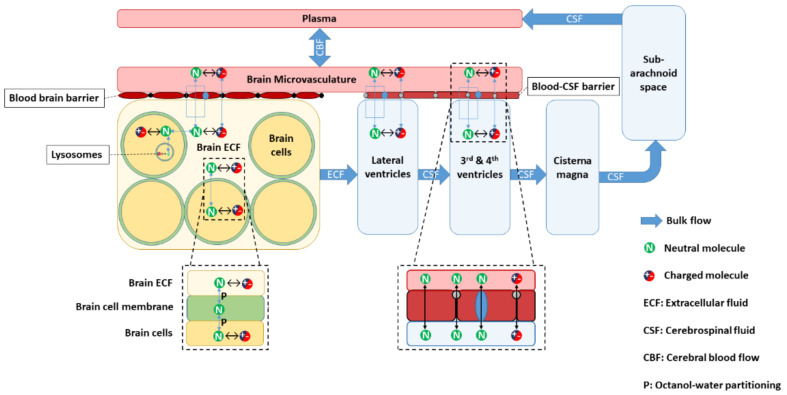
LeiCNS-PK3.0 model structure. The central nervous system (CNS) model connects to the plasma via cerebral blood flow. LeiCNS-PK3.0 accounts for the brain and cerebrospinal fluid (CSF) compartments, the presence of the blood–brain barrier (BBB) and blood–CSF barriers, drug transport across the barriers and within the CNS, and physiological process such as non-specific binding and the effect of pH on drug ionization and on its passive transport.

The “Leiden CNS PBPK predictor v3.0” or LeiCNS-PK3.0 (Figure 1 and Figure S1 in Supplementary Materials) is a CNS PBPK model that adequately predicts the PK of small drug molecules in the CNS of rats and humans on the basis of exclusively plasma PK, drug physicochemical, CNS physiological, and in vitro information [10,12,13]. The LeiCNS-PK3.0 model accounts for the different CNS physiological compartments such as the brain microvasculature, brain_ICF_ and brain_ECF_, lysosomes, and cerebrospinal fluid (CSF) compartments (such as lateral ventricles, third and fourth ventricles, cisterna magna, and subarachnoid space, including the lumbar CSF region). Different drug transport modes within the CNS are represented including drug transport by paracellular, transcellular, and active transport across the BBB and blood–CSF barrier (BCSFB) and by bulk fluid flow from the brain_ECF_ along the CSF compartments back to the plasma. Moreover, the physiological processes that affect intra-brain unbound drug distribution are accounted for, such as brain tissue non-specific binding and the effect of CNS pH on drug ionization.

In general, changes in BBB properties and CNS physiology are common in CNS diseases, as well as in aging or other conditions, but the impact of some of these processes is often overlooked when investigating brain PK in such conditions. These include brain_ECF_ volume, of which the fraction is doubled during sleep and anesthesia [14] and declines with aging [15]; the BBB tight junctions’ paracellular pore radius (para_radius_) that increases for example in Alzheimer’s disease [16], with aging [17], and in traumatic brain injury [18]; CBF that declines for example in Alzheimer’s disease [19], with aging [20], and anesthesia [21]; and pH_ECF/ICF_ that declines for example in traumatic brain injury [22], brain ischemia [23,24], and with aging [25].

In this paper, we use LeiCNS-PK3.0 to explore the effect of the pathophysiological changes of: CBF, para_radius_, brain_ECF_ volume, pH_ECF_, and pH_ICF_ on BBB transport and intra-brain distribution of 46 small drugs of different physicochemical properties.

## 2. Materials and Methods

### 2.1. LeiCNS-PK3.0 Model

This simulation study was performed using LeiCNS-PK3.0 (Figure 1 and Figure S1 in Supplementary Materials) and human CNS physiological parameters (Table 1) [12]. A virtual one-compartment plasma PK model was used as input to the CNS model, with plasma clearance of 297 L/h and a central compartment volume of 108 L. The drug dose was 1 g, which was administered as intravenous infusion over 15 min. The fixed plasma PK model and dosing regimen were used for all investigated drugs, thus solely focusing on the impact of CNS parameters changes on brain_ECF_ and brain_ICF_ PK. More information on the model buildup and the associated equations can be found at [10,12,13].

### 2.2. Drug Parameters

The physicochemical properties of the 46 small drugs (Table 2 and Table S1) in this study were available from the Drugbank database release version 5.1.7 (go.drugbank.com) [26]. These drugs have distinct physicochemical properties such as molecular weight (Mwt: 150–500 g/mol), lipophilicity (logP: −3.7–4.3), acid/base ionization constants (pk_a_: 3–16/pk_b_: −9–10) and different affinities to active transporters. We included calculated pk_a/b_ values from CHEMAXON [27] and included calculated lipophilicity from the ALOGPS method [28], unless experimental octanol–water portioning values were reported.

Active transport across the BBB was described using Kp_uu,BBB_ values (Table 2 and Table S1), which were calculated from rat microdialysis plasma and brain_ECF_ drug concentrations [12,29,30,31]. Then, these were translated to predict human BBB active transport as described in [10], taking into consideration the interspecies difference in protein expression [32,33,34,35,36] of the four main BBB active transporters: P-glycoprotein (p-gp), multi-drug-resistant protein-4 (MRP4), breast cancer resistance protein (BCRP), and organic anionic transporter 3 (OAT3). The protein expression of other relevant transporters at the BBB such as MRP1 was assumed the same in rats and humans, due to the absence of quantitative information on the difference of protein expression in rats and in humans [32,33,34,35,36,37]. Information on drugs affinity to a certain transporter was available from Drugbank [26]. The factors used for the rat-to-human translation are summarized in Table S2. Differences in transporters functionality, which is distinct of expression [38], in rats and humans [39,40,41] were not accounted for. This interspecies difference is not attributed to the transporter per se, but rather to the combination of the drug and the transporter. Given both the scarcity of transporter functionality information in the literature and the goal of the current study, rat-to-human translation was based on differences in expression only. Kp_uu,BCSFB_ values (Table 2 and Table S1), which represent active transport across the BCSFB, were either available from the literature or assumed the same as Kp_uu,BBB_.

### 2.3. Selection of Pathophysiological Parameters Values

The CNS parameters investigated in this study were CBF, para_radius_, Brain_ECF_ volume, pH_ECF_, and pH_ICF_. The changes in the parameters values were selected to reflect their values in CNS diseases. Parameters were changed based on literature values as follows: CBF by 70% [42] and 150% [21]; para_radius_ by 50% and 500% [43]; brain_ECF_ volume by 70% and 150% [14,15]; pH_ECF_ to 5 and 8 [23]; and pH_ICF_ to 6 and 7.6 [24,44].

### 2.4. LeiCNS-PK3.0 Simulations and Data Analysis

LeiCNS-PK3.0 simulations were observed over 600 min for all drugs. For low transcellular permeability drugs such as methotrexate and atenolol, brain_ICF_ PK were incomplete, i.e., it had not reached C_max_ after 600 min, and the observation time was extended to 20,000 min (results not shown). LeiCNS-PK3.0 simulations were performed using RxODE version 0.9.2-0 [45] using LSODA (Livermore Solver for Ordinary Differential Equations) Fortran package and R version 4.0.3 [46].

LeiCNS-PK3.0 simulation results were evaluated by comparing the different PK at brain_ECF_ and brain_ICF_ of different parameters values. In addition, heatmaps were generated to reflect the magnitude of change of C_max_, T_max_, AUC_0–T_, Kp_uu,BBB_, and Kp_uu,cell_. AUCs were calculated using the R package PKNCA version 0.9.4.

Kp_uu,BBB_ and Kp_uu,cell_ were calculated as follows [1]:

$${\mathrm{Kp}}_{\mathrm{uu},\mathrm{BBB}}=\frac{{\mathrm{AUC}}_{0-\infty ,\mathrm{ECF}}}{{\mathrm{AUC}}_{0-\infty ,\mathrm{MV}}}$$

$${\mathrm{Kp}}_{\mathrm{uu},\mathrm{cell}}=\frac{{\mathrm{AUC}}_{0-\infty ,\mathrm{ICF}}}{{\mathrm{AUC}}_{0-\infty ,\mathrm{ECF}}}.$$

For AUC_0–∞_, the elimination rate constant was calculated from the terminal elimination phase and was used to extrapolate the concentration–time curve to time infinity.

Two-fold change was calculated to reflect the effect of changing one parameter on PK parameters; a value of 1 reflects a two-fold change.

$$\mathrm{Two}-\mathrm{fold}\;\mathrm{change}=\log_{2}\left(\frac{\mathrm{PK}.{\mathrm{params}}_{\Delta =x}}{\mathrm{PK}.{\mathrm{params}}_{\Delta =1}}\right)$$

where PK.params_∆=x_ and PK.params_∆=1_ represent the calculated PK parameters (C_max_, T_max_, AUC_0–T_, Kp_uu,BBB_, and Kp_uu,cell_) at x-fold altered and physiological CNS parameters, respectively.

## 3. Results

**Figure 2:**
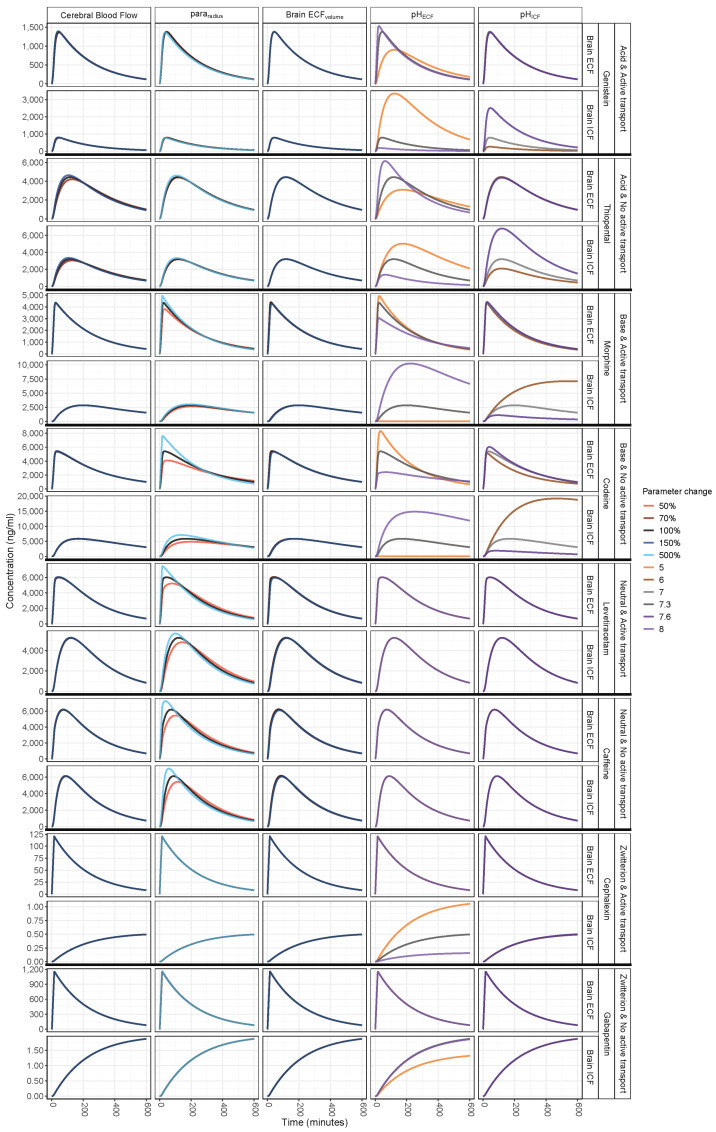
Simulated concentration–time profiles of selected drugs at physiological and pathophysiological values of CBF, tight junction paracellular pore radius (para_radius_), brain_ECF_ volume, pH_ECF_, and pH_ICF_. Para_radius_ affected the rate and extent of passive drug transport across the BBB, pH_ECF_ and pH_ICF_ affected the brain_ECF_ and brain_ICF_ unbound drug concentration-time profile (PK), whereas cerebral blood flow and brain_ECF_ volume had a very modest (if any) effect. The fixed plasma PK used excludes the involvement of plasma PK in the observed changes.

**Figure 3:**
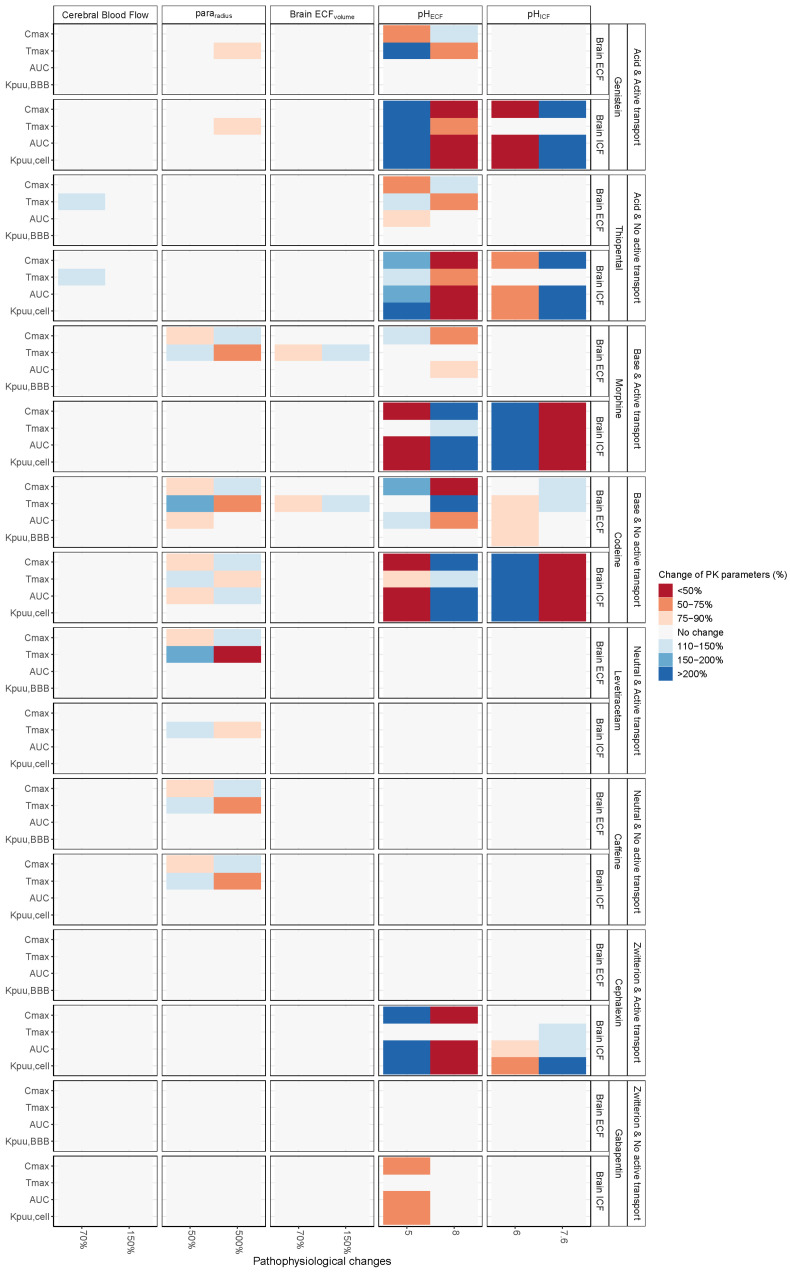
Heatmaps summarizing the effect of pathophysiological changes of CBF, tight junction paracellular pore radius (para_radius_), brain_ECF_ volume, pH_ECF_, and pH_ICF_ on brain pharmacokinetic parameters: C_max_, T_max_, AUC, Kp_uu,ECF_, and Kp_uu,cell_. C_max_ and T_max_ define the rate of BBB drug transport, while AUC and Kp_uu_ define the extent of drug transport. Effect of pathophysiological changes remain drug (class) specific. Similar to the concentration–time profiles, para_radius_, pH_ECF_, and pH_ICF_ had a profound effect on brain pharmacokinetics compared to the minor effect of cerebral blood flow and brain_ECF_ volume. The fixed plasma PK used excludes the involvement of plasma PK in the observed changes.

The simulated impact of pathophysiological changes of CBF, para_radius_, brain_ECF_ volume, pH_ECF_, and pH_ICF_ on PK at brain_ECF_ and brain_ICF_ are displayed for selected drugs in Figure 2 and for all drugs in Figure S2. The associated heatmaps, Figure 3 and Figure S3, reflect the changes in the BBB drug transport rate via C_max,_ and T_max_ and extent via AUC_0–T_, Kp_uu,BBB_, and Kp_uu,cell_. As plasma PK was fixed, any role of plasma in the observed changes is eliminated. The changes of CBF and brain_ECF_ volume affected the rate but not the extent of BBB drug transport, whereas changes in pH_ECF_, pH_ICF_, and para_radius_ affected both the rate and extent of BBB drug transport.

### 3.1. Increased Passive Transport via Widened Para_radius_

Figure 2 and Figure 3 (2nd column) demonstrate that the impact of a changed para_radius_ on BBB drug passive transport varied according to the drug lipophilicity, ionization at physiological pH, and affinity to active transporters. Of interest, a five-fold increase in para_radius_ resulted in a decrease in the extent of BBB transport of risperidone, paliperidone, and omeprazole, as demonstrated by a decrease in AUC_0–T,ECF_ and in Kp_uu,BBB_.

### 3.2. pH_ECF_ and pH_ICF_ are Key Factors of Drug Distribution in Brain_ECF_ and Brain_ICF_

Figure 2 and Figure 3 (4th and 5th columns) show the influence of pH changes on the rate and extent of drug transport across the BBB and across the brain cell membranes. A pH increase in a given compartment generally resulted in a faster rate and increased the extent of acidic drug transport and a slower rate and decreased the extent of the basic drug transport into that compartment, and vice versa. The rate and extent of drug transport in the adjacent compartment were affected in an inverse fashion. For amphoteric drugs, the effect of pH on their transport rate and extent was relative to the ionization constants of their strongest acidic and basic groups. As expected, pH changes had no effect on drugs that are neutral at the physiological pH range.

### 3.3. Brain_ECF_ Volume and CBF Have a Very Modest Effect on Rate of BBB Drug Transport

Figure 3 (1st and 3rd columns) display only a T_max_ increase of <50% associated with a 50% increase of brain_ECF_ volume, while a slight T_max_ decrease of <25% was noticed with a 30% decrease of brain_ECF_ volume. With regard to CBF, a 30%-decrease resulted in a <50%-delay of T_max_, whereas a 50%-increase resulted in a <25%-earlier T_max_. These effects were associated with neutral drugs of relatively higher net BBB influx.

## 4. Discussion

LeiCNS-PK3.0 simulations have demonstrated the drug-dependent effect of pathophysiological changes of para_radius_ on the rate and extent of BBB passive drug transport, and of pH_ECF_ and pH_ICF_ on the PK of brain_ECF_ and brain_ICF_.

LeiCNS-PK3.0 allows the prediction of PK in the less accessible brain tissue and the potential PK changes associated with diseased conditions. LeiCNS-PK3.0 predictions are based explicitly on human CNS physiological parameters available from the literature, drug physicochemical parameters available from Drugbank database [26], and translated data from in vitro and preclinical studies. Thus, LeiCNS-PK3.0 overcomes the technical and ethical limitations of experimental approaches, such as the invasiveness of microdialysis, inability to differentiate parent drug and metabolite with imaging techniques, and the inaccurate lumbar CSF surrogacy to brain PK [12,102].

Paracellular passive diffusion across the BBB tight junction pores is especially critical for small, hydrophilic drugs, whose transport across the lipophilic membranes of BBB endothelial cells is limited, although this paracellular route represents about 0.004% of BBB surface area [12]. Increased passive transport via this route has been reported after BBB opening with hyperosmotic mannitol, where the brain exposure of atenolol [43] and methotrexate [103] increased by about 3- and 5-folds, respectively. BBB opening and widening of para_raduis_ after hyperosmotic mannitol were confirmed in the latter study using electron microscopy [103]. In CNS diseases, BBB permeability to drug transport across the paracellular route increases (Table 3). The impact of increased para_radius_ on passive transport across the BBB is rather dependent on the balance between passive transcellular and passive paracellular drug transport, the difference in pH between the compartments, and the contribution of active transporters to influx or efflux BBB transport (Table 2 and Table S1 in Supplementary Materials). An increase of passive paracellular transport will generally result in Kp_uu,BBB_ closer to unity [1]. Drugs that are heavily reliant on the transcellular route or on active transport are less sensitive to changes in para_radius_. Drug physicochemical properties might also play a role, as the three drugs, whose BBB transport extent was affected, were lipophilic bases.

PH changes are relevant for drugs with pk_a_ < 9 and/or pk_b_ > 3, which ionize at the physiological pH range of 5–7.4, as the ionized drug species do not cross the transcellular route or cell membrane as assumed in LeiCNS-PK3.0 and are thus trapped in brain_ICF_ and lysosomes or can escape brain_ECF_ via the paracellular route and with ECF bulk flow [12]. A consequence of the trapping assumption is that the difference in pH across a membrane will result in unequal drug partitioning across the membrane. This phenomena has been overlooked in several studies where changes in brain_ECF_ PK due to traumatic brain injury were attributed to a reduction of active transport [5,10] and increase para_radius_ [5,10,104], but not to pH_ECF_. The results of our simulation strongly suggest that pH changes in CNS disease might play a bigger role in defining disease brain PK than previously conceived.

The impact on brain PK due to changes in para_radius_, pH_ECF_, and pH_ICF_ during traumatic brain injury (TBI), Alzheimer’s disease (AD), brain malignancies, cerebral ischemia, and epilepsy has been explored, as guided by LeiCNS-PK3.0 simulations. The pathophysiological changes of the three parameters in these CNS diseases are listed in Table 3. Quantitative information on para_radius_ values in the different diseases are not always reported, and therefore, BBB permeability as an indirect measure of para_radius_ was used.

Microdialysis studies in TBI patients have shown that brain_ECF_ PK is different in the healthy versus injured brain tissue. In two independent studies, morphine PK was higher in the injured than in the healthy brain tissue of adult [104] and pediatric TBI patients [4]. In addition, cyclosporine brain_ECF_ PK might change in TBI patients [105]. In TBI patients, changes occur to pH_ECF_, pH_ECF_, and to para_radius_; the magnitude of change and time course of these parameters may differ according to trauma type: focal vs. diffuse TBI or close-head vs. open-head injury. In TBI patients, pH_ECF_ and pH_ICF_ decline to 7 [22] and 6.9 [106], respectively. PH measurements in TBI mice suggest a biphasic change of pH, which resolves after two hours, while in TBI patients, pH showed a resolution to normal values after about 10 days [22,106]. PH_ICF_ changes are of minor impact on traumatic brain PK. However, pH_ECF_ changes due to TBI might impact brain PK of drugs with pk_a_ < 8 and pk_b_ > 6, respectively. The BBB opening is another feature of TBI, as evidenced by the decrease in tight junction protein expression mainly claudin-5, occludin, and ZO-1 and an increase in BBB permeability to small and medium (0.1–10 kDa) and large molecules (up to 160 kDa) [107,108,109] in TBI mice. BBB opening and increased permeability resides up to the first 96 and 24 h post-injury for small and large molecules, respectively [107,108,109]. A wide range of CNS-acting medications are used to manage TBI patients including analgesics (e.g., acetaminophen, morphine), anticonvulsants (e.g., gabapentin and carbamazepin), neuroprotective agents (e.g., cyclosporine), etc. LeiCNS-PK3.0 simulations at altered para_radius_ and pH_ECF/ICF_ have shown that the CNS PK of some of these drugs are potentially affected by these changes. An increase in para_radius_ resulted in an increase in brain_ECF_ C_max_ of morphine. Changes in pH_ECF/ICF_ might affect the PK of morphine (pkb = 9.1) and gabapentin (pk_a_ = 4.6, pk_b_ = 9.9). Combining the simulation results and literature findings on TBI pathophysiology and in vivo TBI PK suggests that brain PK may change due to pH and para_radius_, particularly during the first 48 h after the injury.

Brain PK is potentially altered in epilepsy. Brain PK of phenytoin was lower in epileptic compared to control rats; the difference was accounted for by the increased p-gp expression in epileptic rats [110]. Brain PK of phenytoin increased following a seizure when p-gp expression was suppressed with nimodipine, implying a potential role of the BBB opening in altering phenytoin PK. Postmortem studies in rats and humans have demonstrated an increased BBB permeability to albumin and Evan’s blue (Mwt = 69 kDa) following an epileptic seizure [111], which persisted in rats up to 1 week after the seizure [111]. Epileptic seizures result as well in a decrease in pH_ECF_ by 0.5 units, which returns to normal values at a slower rate than pH_ICF_, which declines by about 0.3 pH units and is corrected within 20 min following seizure [112]. These changes in pH are expected to impact drugs with pk_a_ < 8 and pk_b_ > 6, respectively. Our simulations included antiepileptic drugs such as phenytoin, diazepam, carbamazepine, levetiracetam, and gabapentin. Of these drugs, only levetiracetam was sensitive to changes in para_radius_, while gabapentin (a zwitterion, pk_a_ = 4.6 and pk_b_ = 9.9) PK in brain_ICF_ was sensitive to changes in pH_ECF_. Phenytoin PK changes remains interesting, as despite experimental evidence of the importance of the passive transport route [110], LeiCNS-PK3.0 simulations showed no sensitivity to para_radius_ changes. It is worth mentioning that in vitro studies using human- and mouse-derived p-gp have concluded that phenytoin is actively transported in rodents but not in humans [113].

Glioma patients and sarcoma-laden rats showed higher methotrexate brain_ECF_ PK compared to controls [7]. Cyclophosphamide brain_ECF_ PK, on the contrary, was lower in tumor-bearing vs. non-tumor-bearing mice [31]. Brain tumors affect BBB permeability as demonstrated by the 8-fold increase in para_radius_ in rats with a malignant glioma [114], which was measured with gadolinium-labeled nanoparticles of increasing size. In addition, the pH_ECF_-to-pH_ICF_ ratio is reversed in brain tumors, as pH_ECF_ decreases to 6.7, whereas pH_ICF_ increases to 7.3 [115,116]. This will result in the change in PK and drug partitioning between brain_ECF_ and brain_ICF_ [100], which is indicated by our Kp_uu,cell_ values (Figure 3 and Supplementary Figure S3), particularly for drugs with acidic and basic groups of pk_a_ and pk_b_ of <8 and >6, respectively. LeiCNS-PK3.0 simulations of the chemotherapeutic drugs, cyclophosphamide and methotrexate, showed a decline of T_max_ due to increased para_radius_, while only methotrexate (pk_a_ = 3.4) PK at brain_ECF_ and brain_ICF_ PK was sensitive to pH_ECF_ and pH_ICF_ changes.

Profound changes in para_radius_ and pH_ECF/ICF_ during cerebral ischemia suggest a change in ischemic brain PK; however, evidence of such changes are not available in the literature. The BBB permeability of gadolinium (Mwt = 590 Da) and Evan’s blue increased in a rat model of cerebral ischemia–reperfusion injury, and this increase resided for 4 weeks for gadolinium and for 3 weeks for Evan’s blue [117]. In addition, cerebral ischemia is associated with a 4-h severe brain acidosis, where the pH_ECF_ declines to 5.9 [118], while pH_ICF_ declines to 5 [23,24,119]. This drastic pH change will result in altering the PK of both acidic (pk_a_ < 8) and basic (pk_b_ > 4) drugs.

Disease translation pharmacokinetic modeling is crucial for accurate predictions of drug effect, but it is challenging particularly for CNS diseases that are progressive, with yet unraveled pathophysiology mechanisms and scarce (pre)clinical data for model validation, not mentioning the ethical concerns in this sensitive yet critical research field. Thus, predicting a disease-specific PK at brain target sites requires a holistic approach such as PBPK modeling that accounts for both drug and (patho)physiology. In this manuscript, we applied our CNS PBPK model, LeiCNS-PK3.0, to predict the impact of altering one CNS parameter at a time on brain PK. LeiCNS-PK3.0 can also be used to predict a disease-specific PK in different regions of the CNS. This will require accounting for all disease-specific pathophysiological changes such as changes in tissue composition and non-specific binding [120], tissue volumes [121], active transporter expression and functionality [38], pH changes, CSF-related changes [12], etc. and their time course, i.e., deteriorating vs. healing. Such information is not always available from humans, and therefore, translating information on CNS pathophysiology from preclinical species is indispensable. Plasma PK acts as input to LeiCNS-PK3.0, and therefore, having the right plasma model from the disease population of interest is a crucial step to accurate CNS PK predictions. Plasma PK might change in CNS diseases compared to a healthy situation due to drug–drug interactions associated with concomitant drug administrations or due to declining liver and kidney functions as seen in elderly and AD patients.

## 5. Conclusions

With LeiCNS-PK3.0 simulations of CNS disease pathophysiology, we demonstrated that the BBB opening might increase the rate and extent of BBB passive transport and that a change of pH_ECF_ and pH_ICF_ can result in altered distribution of unbound drug in brain_ECF_ and brain_ICF_. The impact of those parameters on CNS PK should not be underestimated. It should be noted that our study conclusions remain limited to small drug molecules and may not extend to other drug classes as biologics.

## Figures and Tables

**Table 1:** Human CNS physiological parameters used in LeiCNS-PK3.0.

Parameter		Value	Range	Reference
Volumes (mL)	Total brain	1250	1110–1380	[47,48,49,50]
	Brain extracellular fluid (brain_ECF_)	253 ^1^	217–300	[51,52,53,54,55]
	Brain intracellular fluid (brain_ICF_)	1000 1		calculated
	Brain cell lysosomes (V_LYS_)	12.5 ^2^		[56]
	Brain microvasculature	45 ^3^	37–50	[53,57,58]
	Lateral ventricles	20	11–16	[59,60,61,62,63]
	3rd and 4th ventricles	3	2.3–3.7	[61,62]
	Cisterna magna	1		[64]
	Subarachnoid space	116	110–116	[65,66,67]
Flows (mL/min)	Cerebral blood flow (CBF)	689	644–722	[68,69,70]
	Brain ECF bulk flow	0.2 ^4^		[71]
	CSF flow	0.42	0.28–0.68	[67,72,73,74,75]
Surface areas (cm^2^)	Blood–brain barrier (SA_BBB_)	150,000	140 × 10^3^−360 × 10^3^	[76,77,78,79,80,81,82,83,84]
	Blood CSF barrier (SA_BCSFB_)	15,000 ^5^		[85,86]
	Brain cell membrane (SA_BCM_)	2,666,520 ^6^		[87,88]
	Lysosomes membrane	1,980,260 ^7^		[89,90,91,92,93]
Width (µm)	Blood brain barrier	0.5	0.2–0.4	[81,94]
	Blood CSF barrier			
Number	Total brain cells (N_br,cells_)	1.71× 10^11^ ^8^		[87,88]
Paracellular pore radius (µm)	Blood–brain barrier (para_radius_)	0.0007	0.0007–0.0009	[10,13,95,96]
	Blood CSF barrier	0.0027		[10,13,95]
Effective surface area (%)	BBB Transcellular transport	99.8		[13,97,98]
	BCSFB Transcellular transport	99.8		
	BBB paracellular transport	0.004 ^9^		[10,95]
	BCSFB paracellular transport	0.016 ^9^		
pH	Plasma and brain MV	7.4		[99]
	Brain extracellular fluid (pH_ECF_)	7.3		[100]
	Cerebrospinal fluid	7.3		[101]
	Brain cells (pH_ICF_)	7		[100]
	Brain cell lysosomes	5		[100]

^1^ Volume ratio of Brain_ECF_:Brain_ICF_ is 1:4. ^2^ Calculated as 1.25% (1/80) of brain_ICF_ volume; based on liver lysosomes. ^3^ Calculated as 3.67% of total brain volume. ^4^ Assumed as 50% of CSF bulk flow. ^5^ SA_BCSFB_ = 0.1 * SA_BBB_. SA_BCSFB_ at LV (and TFV) is assumed 50% of SA_BCSFB_. ^6^ SA_BCM_ = SA_cell_^*^ N_br,cells_. Radius_br,cell_ was calculated with Brain_ICF_ volume and N_br,cells_, assuming spherical cells. ^7^ Based on V_LYS_ and mean radius of lysosomes in monkey kidney, rat kidney, and rat neuronal cell (0.1875 µm). ^8^ Based on 1500 gm brain. ^9^ Based on an endothelial cell perimeter of 17 µm.

**Table 2:** Physicochemical properties, active transporters affinities, and BBB transport clearances of selected drugs.

Drug	Mwt	logP	Drug Ion Class	pk_a_	pk_b_	Kp_uu,ECF_	Kp_uu,LV_	Kp_uu,CM_	BCRP	p-gp	OAT3	MRP4	CL_p_	CL_T,ef_	CL_T,in_
Caffeine	194.2	−0.07	Neutral	NA	−0.92	0.96 ^1^	0.96 1	0.96 1	X	-	-	-	48.9	4.28	2.38
Cephalexin	347.4	0.65	Zwitterion	3.26	7.23	0.015 1	0.015 1	0.015 1	-	-	X	-	37.4	2736	<0.01
Codeine	299.4	1.39	Base	13.8	9.19	1 1	1 1	1 1	-	-	-	-	40.1	0.71	0.89
Gabapentin	171.2	1.25	Zwitterion	4.63	9.91	0.13 1	0.13 1	0.13 1	-	-	-	-	51.9	347	<0.01
Genistein	270.2	3.04	Acid	6.55	−5.3	0.04 1	0.041	0.04 1	X	X	-	-	42.3	1557	245
Levetiracetam	170.2	−0.64	Neutral	16.1	−1.6	0.31 1	0.31 1	0.31 1	-	X	-	X	52.0	3.73	0.69
Morphine	285.3	0.87	Base	10.3	9.12	0.23 ^2^	0.23 2	0.23 2	-	X	-	-	41.0	30.2	0.34
Thiopental	242.3	2.85	Acid	7.2	−3	0.9 1	0.9 1	0.9 1	-	-	-	-	44.2	569	508

^1^ [29]; ^2^ [12]; Mwt: molecular weight (g/mol); logP: octanol–water partition coefficient; pk_a_: acid dissociation coefficient; pk_b_: base dissociation coefficient; CL_T,ef_: transcellulr efflux clearance (in mL/min) at BBB; CL_T,in_: transcellulr influx clearance (in mL/min) at BBB; CL_P_: paracellular passive BBB clearance (in mL/min); X: active transporter substrate; p-gp: P-glycoprotein, MRP4: multi-drug-resistant protein-4, BCRP: breast cancer resistance protein, OAT3: organic anionic transporter-3. CL_T,ef_, CL_T,in_, and CL_P_ are calculated as described in [12,13].

**Table 3:** Pathophysiological changes of para_radius_, pH_ECF_, and pH_ICF_ in multiple CNS diseases.

Disease	Parameter	Value	References
Alzheimer’s	BBB permeability	↔ (86–150,000 Da)	[107]
	pH_ECF_	↓ (0.01 pH unit/decade)	[25]
	pH_ICF_		
Brain tumors	para_radius_	↑ (800%)	[114]
	pH_ECF_	↓ (0.6 pH unit)	[115,116]
	pH_ICF_	↑ (0.3 pH unit)	[115,116]
TBI	BBB permeability	↑ (up to 160,000 Da)	[107,108,109]
	pH_ECF_	↓ (0.3 pH unit)	[22]
	pH_ICF_	↓ (0.1 pH unit)	[106]
Ischemia	BBB permeability	↑ (up to 70,000 Da)	[117]
	pH_ECF_	↓ (1.4 pH unit)	[118]
	pH_ICF_	↓ (2 pH unit)	[23,24,119]
Epilepsy	BBB permeability	↑ (albumin and up to 70,000 Da)	[111]
	pH_ECF_	↓ (0.5 pH unit)	[112]
	pH_ICF_	↓ (0.3 pH unit)	[112]

## Data Availability

The data presented in this study are available on request from the corresponding author. The data are not publicly available due to privacy.

## Supplementary Materials

The following are available online at https://www.mdpi.com/1999-4923/13/1/95/s1, Figure S1. Detailed mathematical structure of LeiCNS-PK3.0; Figure S2. Simulated concentration–time profiles of all 46 drugs at physiological and pathophysiological values of CBF, para_radius_, brain_ECF_ volume, pH_ECF_, and pH_ICF_; Figure S3. Heatmaps summarizing the effect of pathophysiological changes of CBF, para_radius_, brain_ECF_ volume, pH_ECF_, and pH_ICF_ on brain pharmacokinetics parameters: C_max_, T_max_, AUC, Kp_uu,ECF_, and Kp_uu,cell_; Table S1. Physicochemical properties and active transporter affinity of all 46 drugs; Table S2. Mean protein expression levels (in fmol/μg total protein) of relevant transporters at the BBB.
