# Supplementary material for: Impact of CNS Diseases on Drug Delivery to Brain Extracellular and Intracellular Target Sites in Human: A “WHAT-IF” Simulation Study
Source: Pharmaceutics. 2021 Jan 13;13(1):95. doi: 10.3390/pharmaceutics13010095 (PMC7828633; doi:10.3390/pharmaceutics13010095)
Supplement: Supplementary file 1 [file pharmaceutics-13-00095-s001.pdf]

pharmaceutics

MDPI

Supplementary Materials: Impact of CNS Diseases on Drug Delivery to Brain Extracellular and Intracellular Target Sites in Human: A "WHAT-IF" Simulation Study

Mohammed A. A. Saleh and Elizabeth C. M. de Lange *

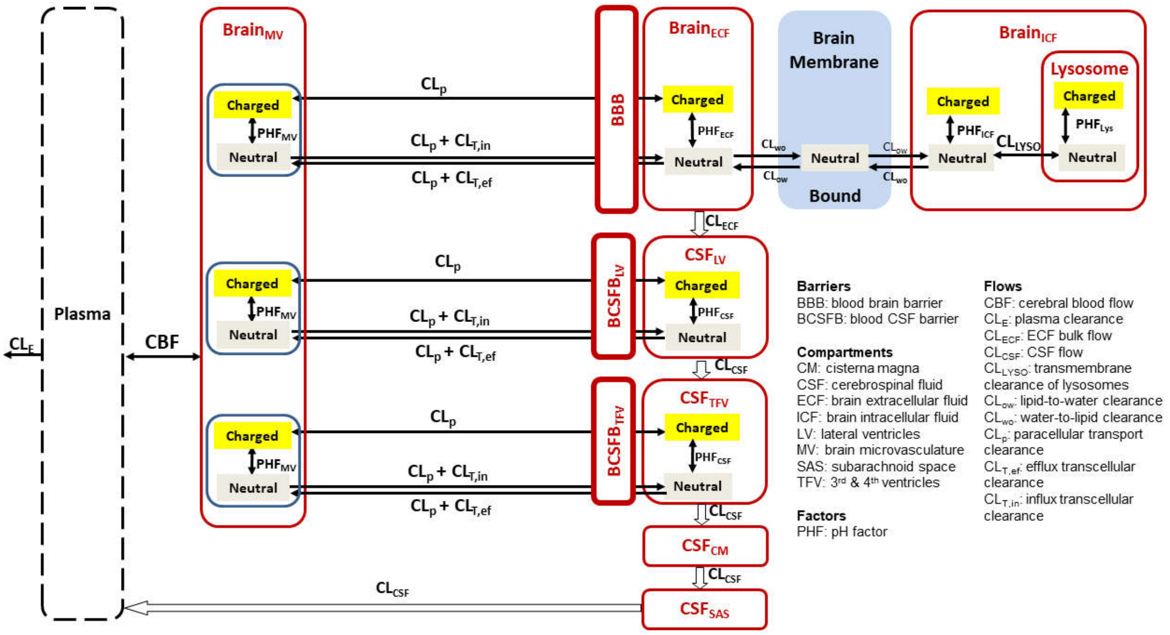

Figure S1. Detailed mathematical structure of LeiCNS-PK3.0.

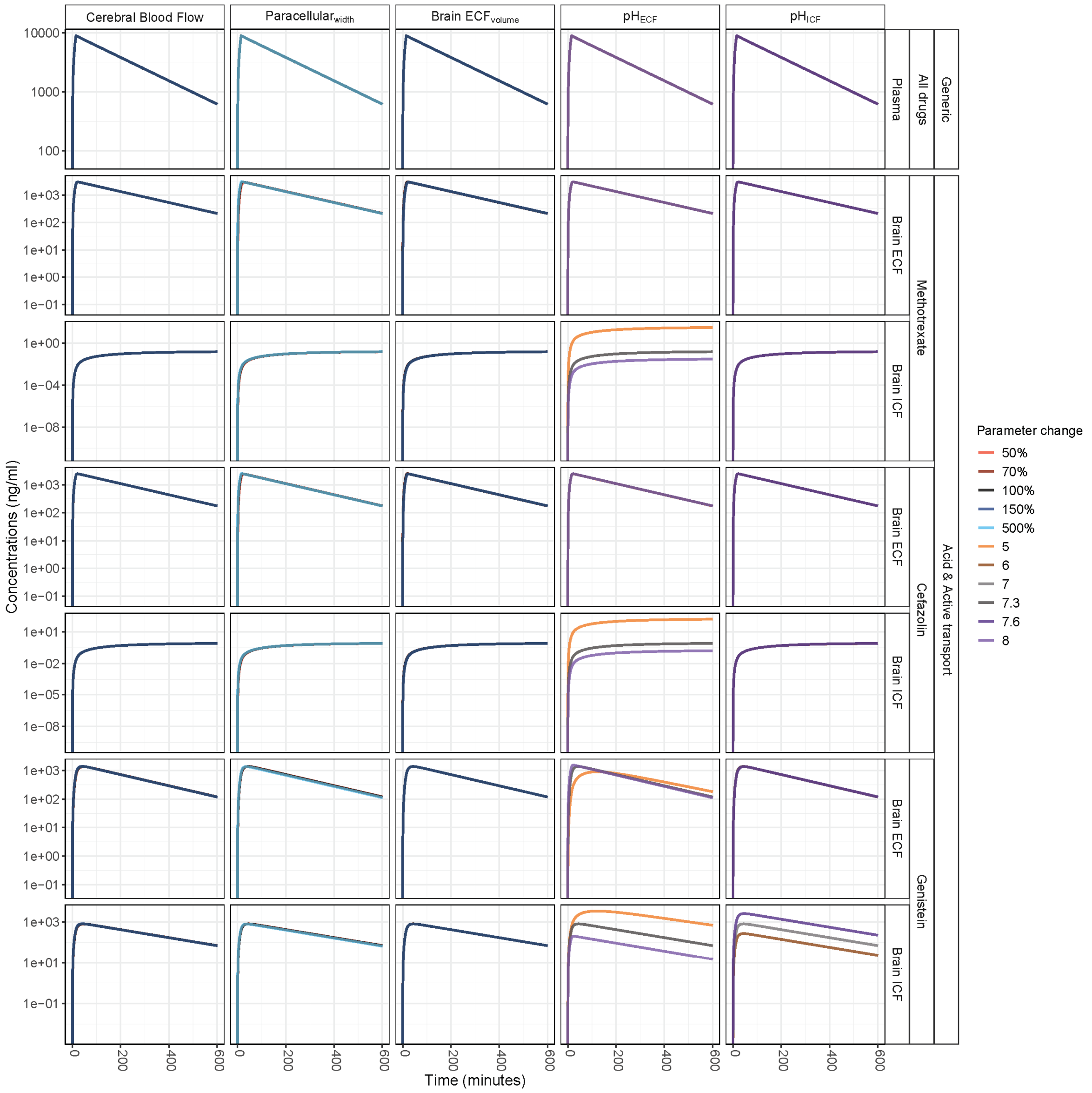

Figure S2. Simulated concentration-time profiles of all 46 drugs at physiological and pathophysiological values of CBF, $para_{radius}$ ($paracellular_{width}$), $brain_{ECF}$ volume, pH_{ECF}, and pH_{ICF}.

Figure S2. Continued

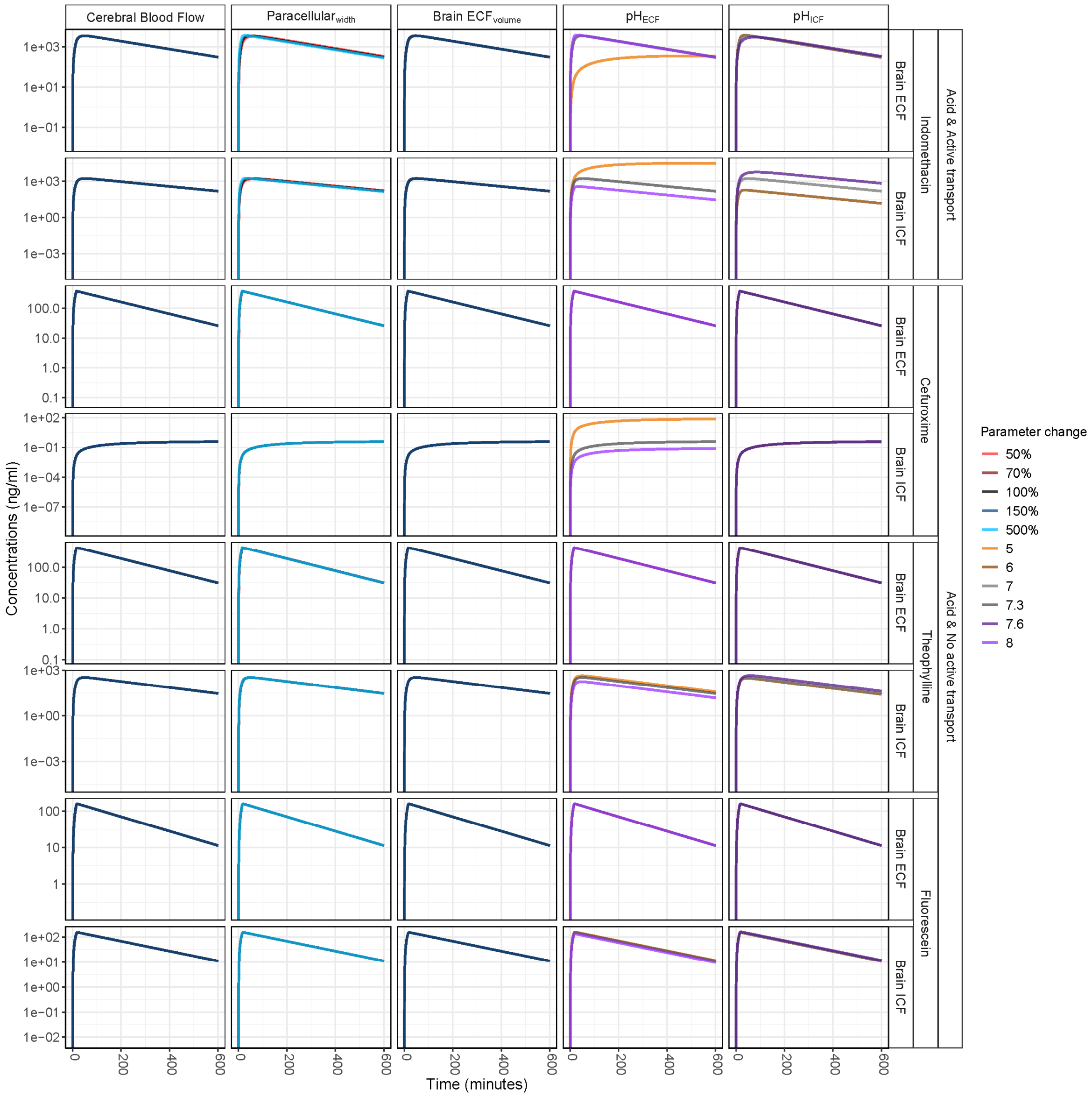

Figure S2. Continued

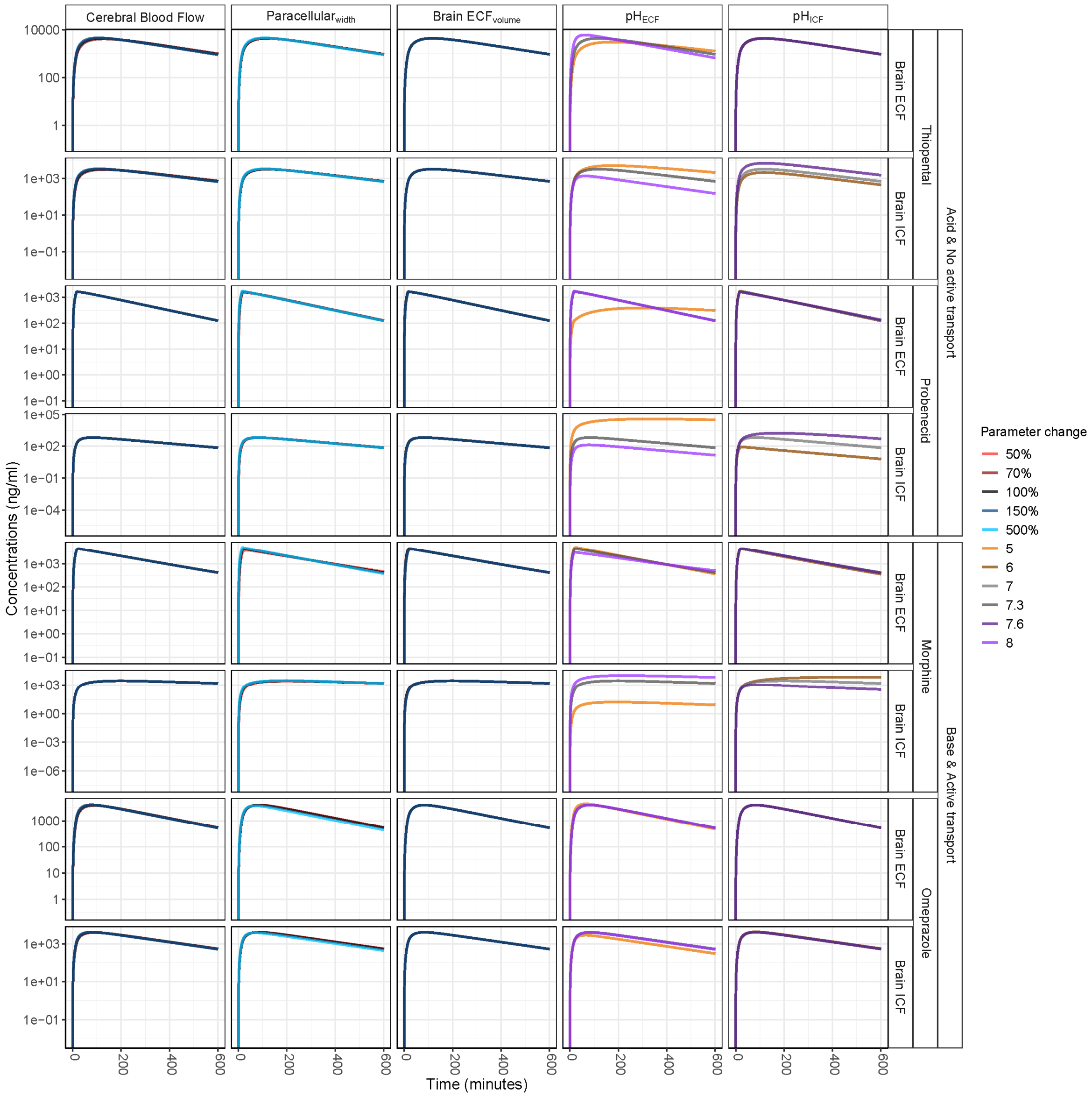

Figure S2. Continued

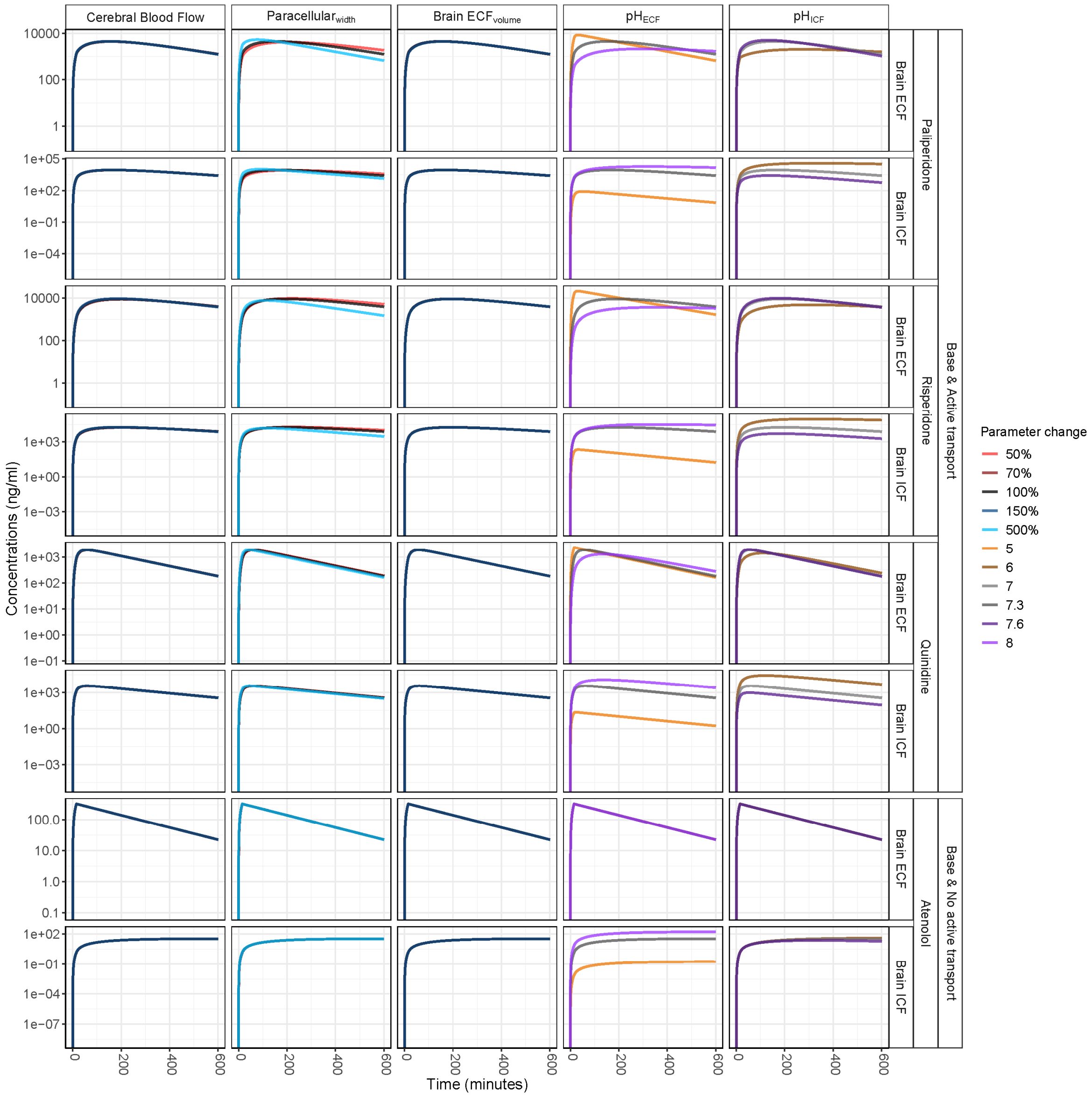

Figure S2. Continued

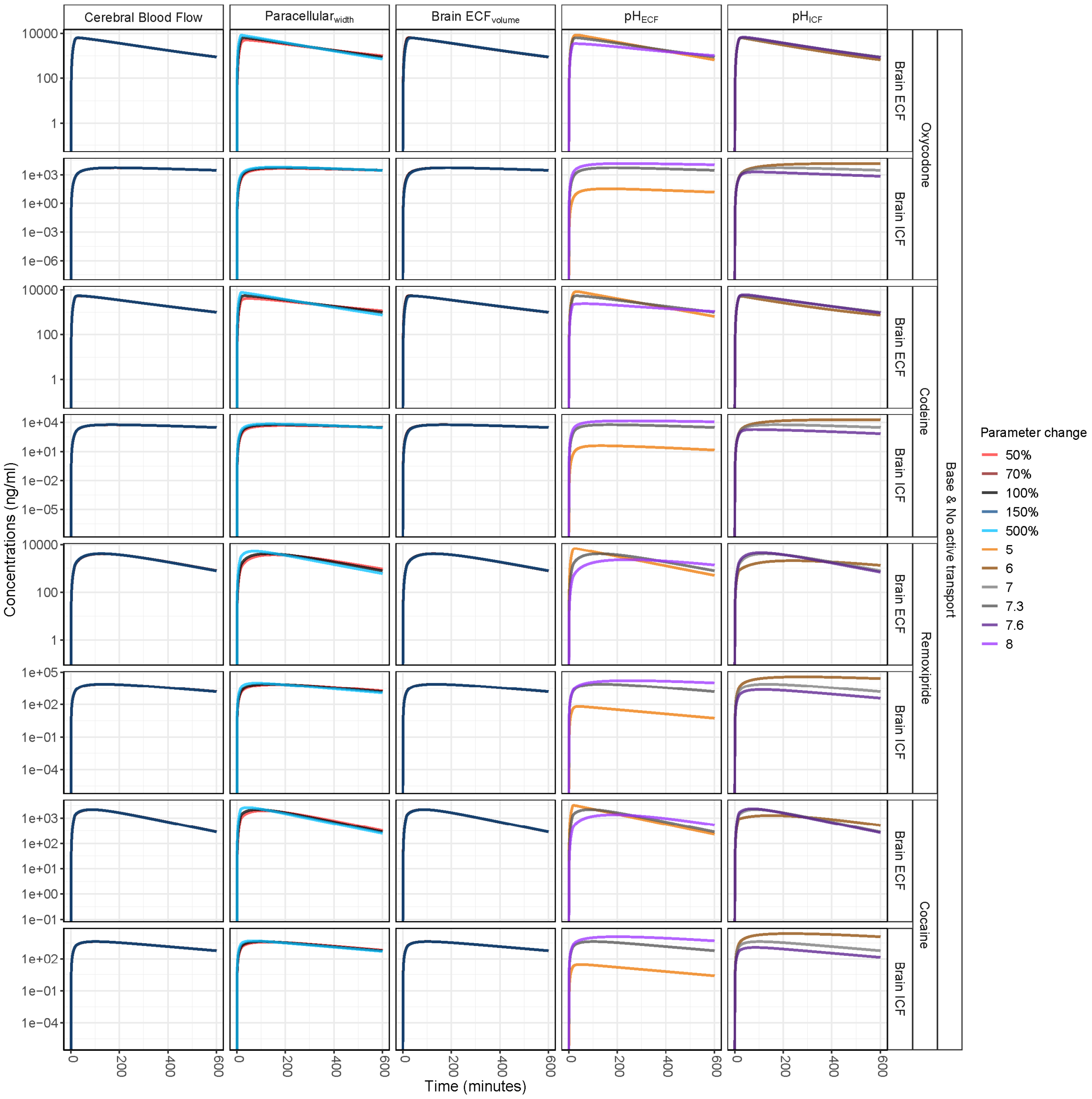

Figure S2. Continued

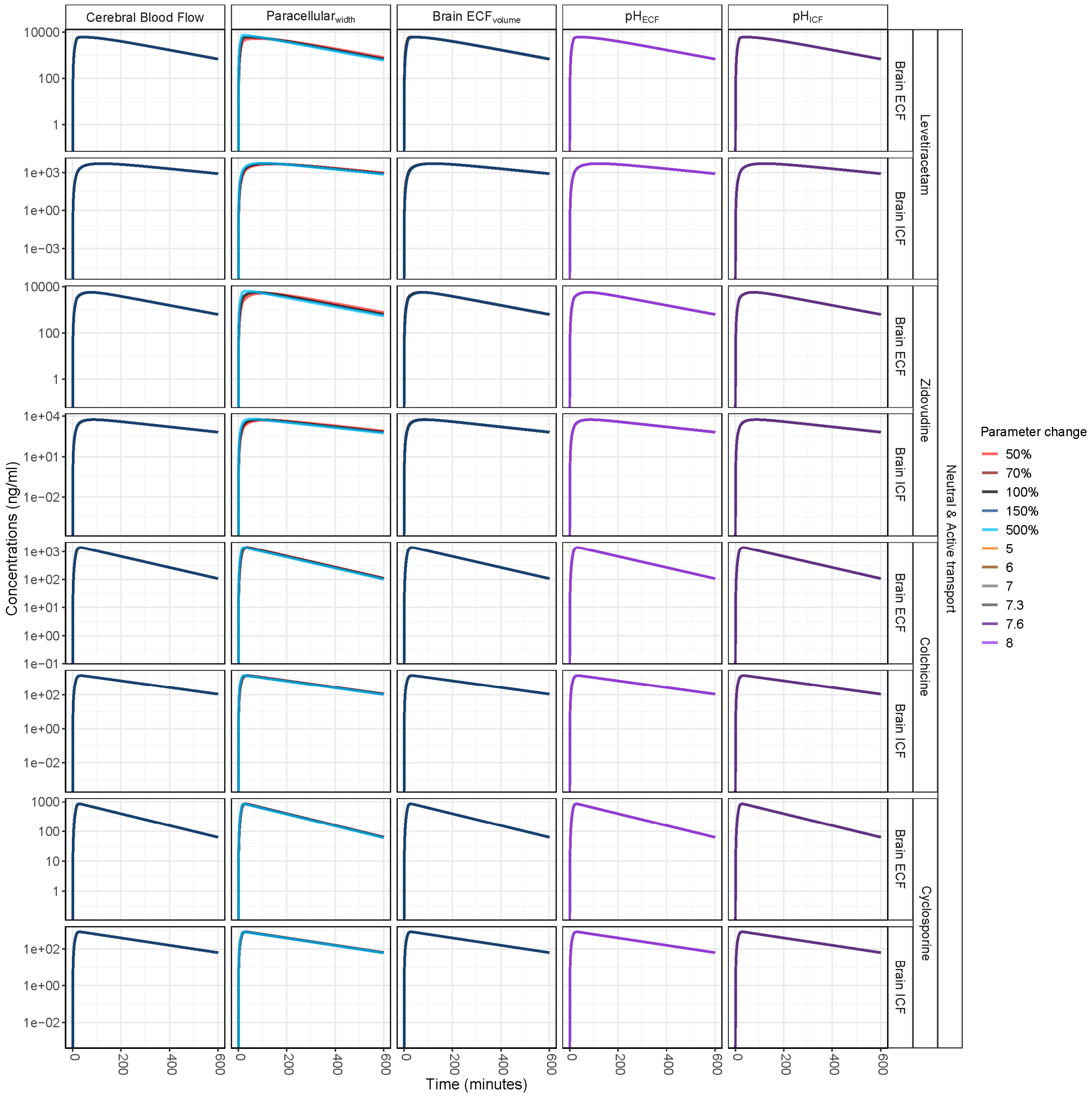

Figure S2. Continued

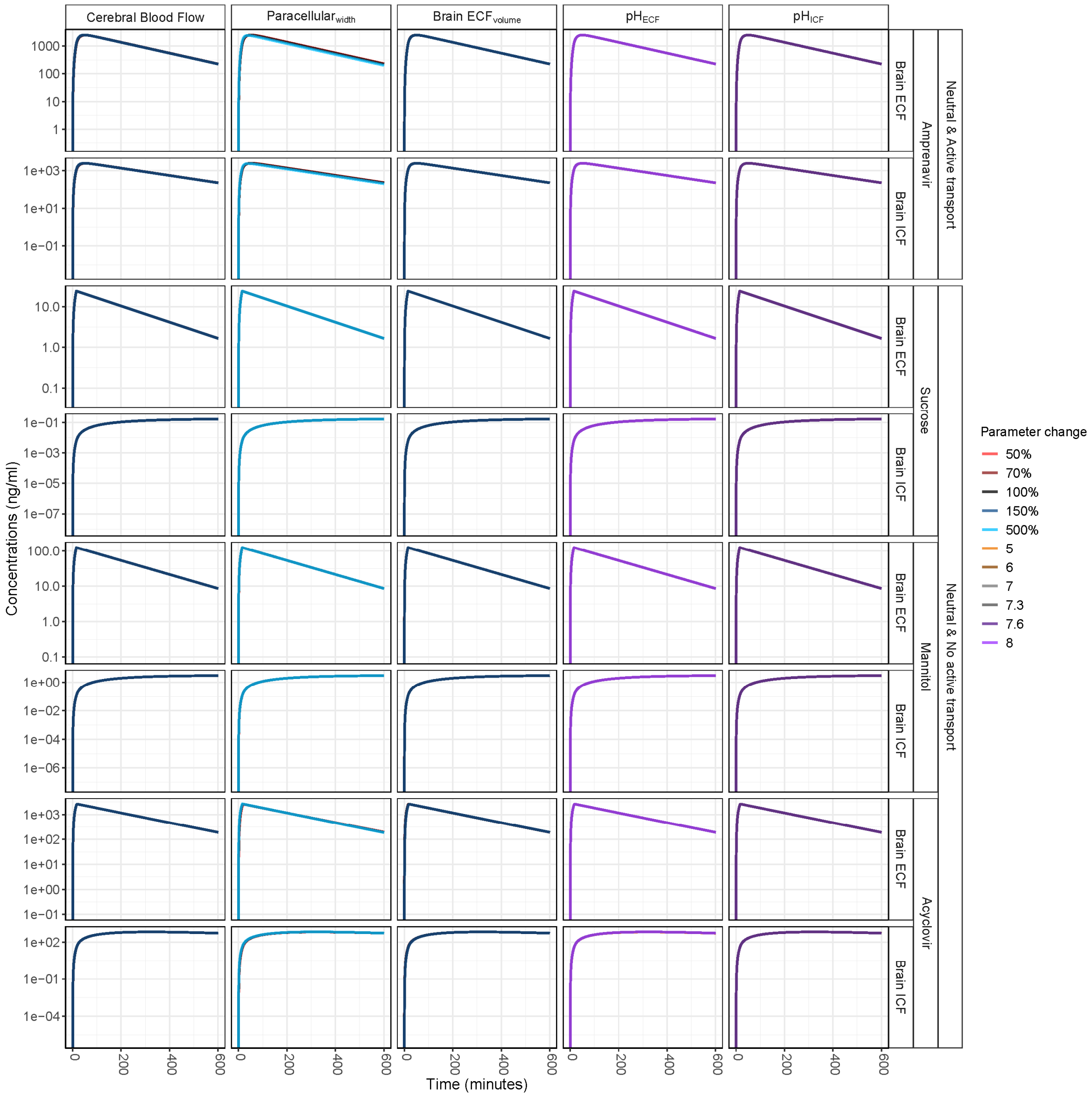

Figure S2. Continued

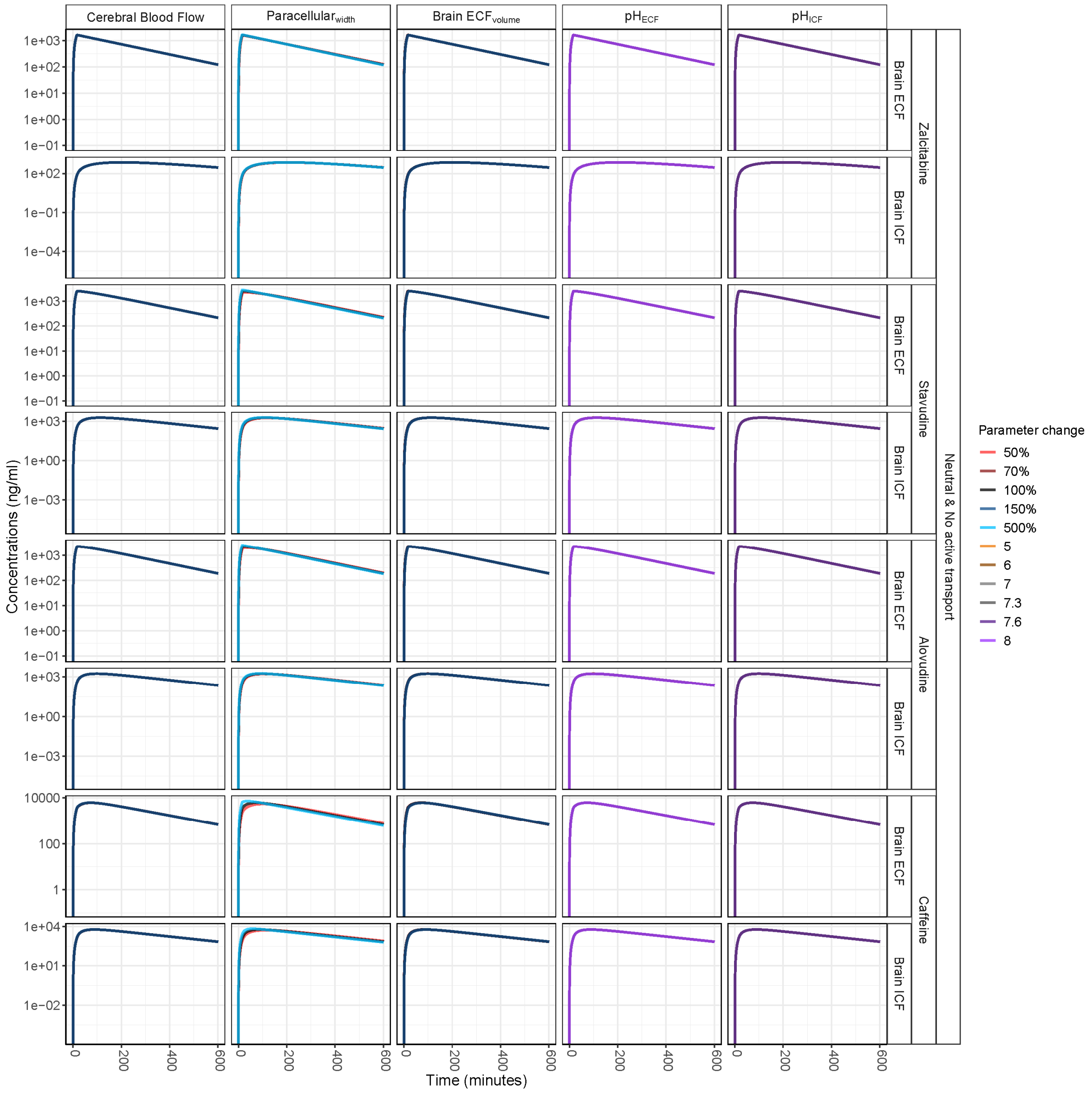

Figure S2. Continued

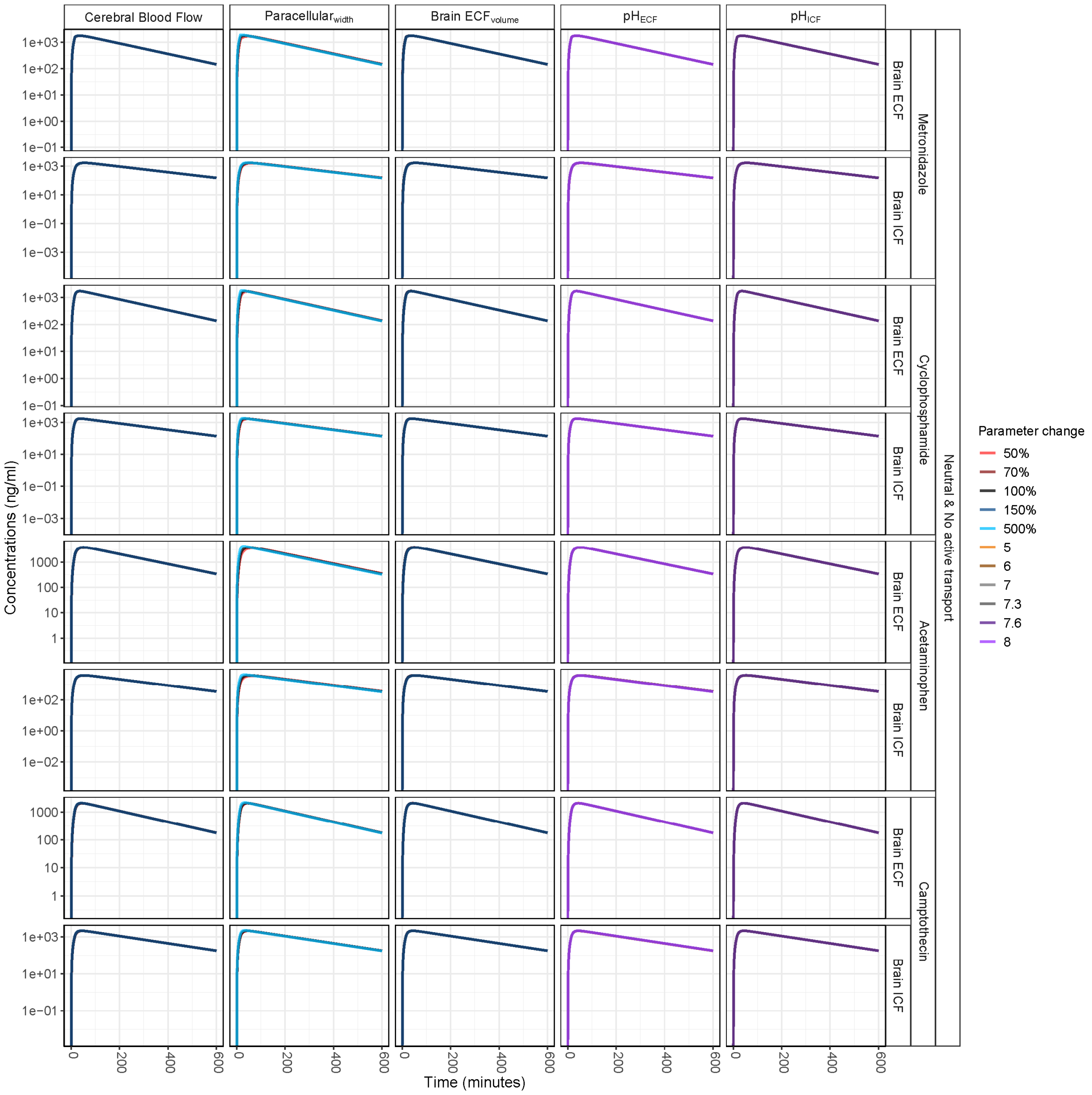

Figure S2. Continued

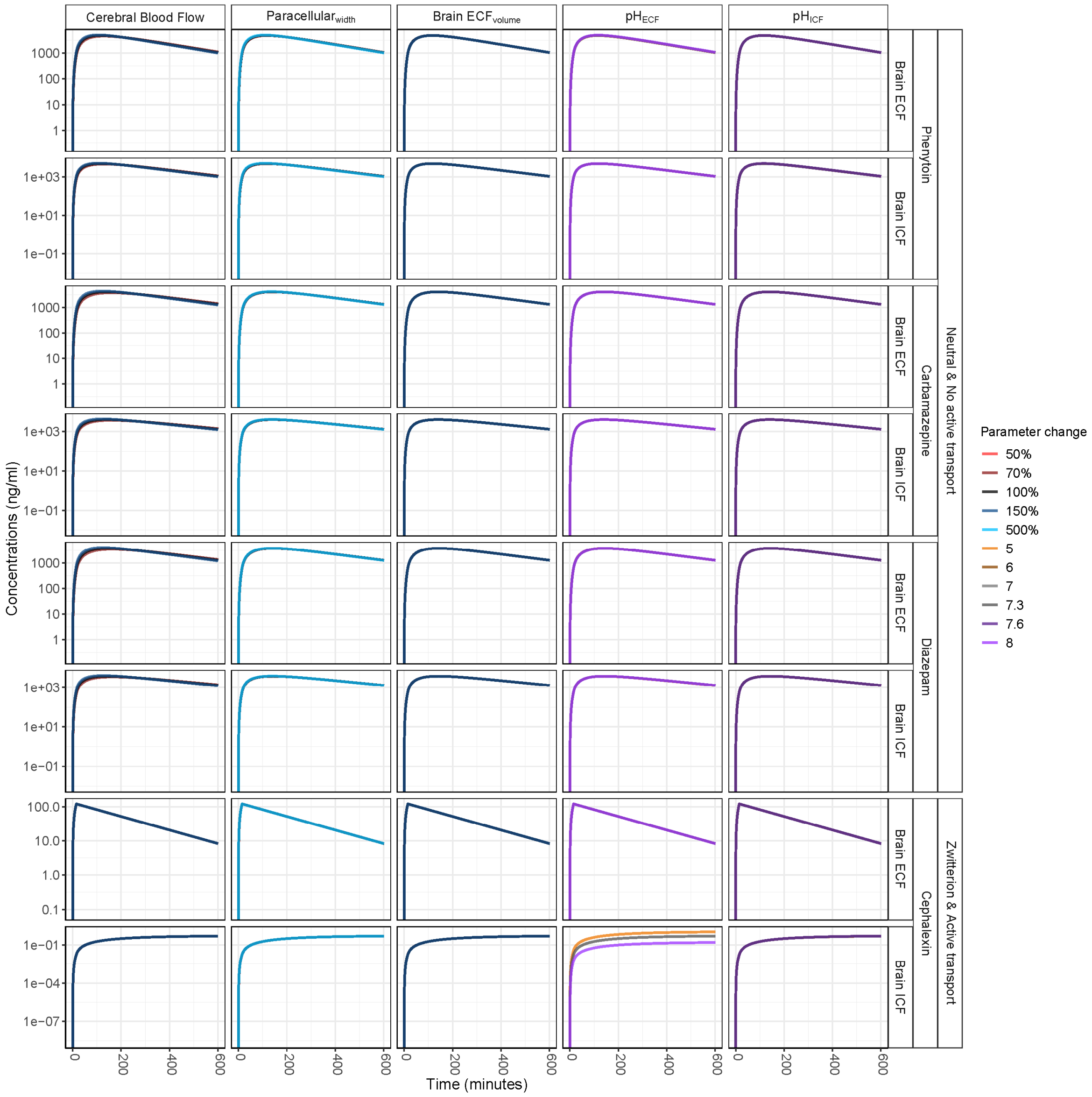

Figure S2. Continued

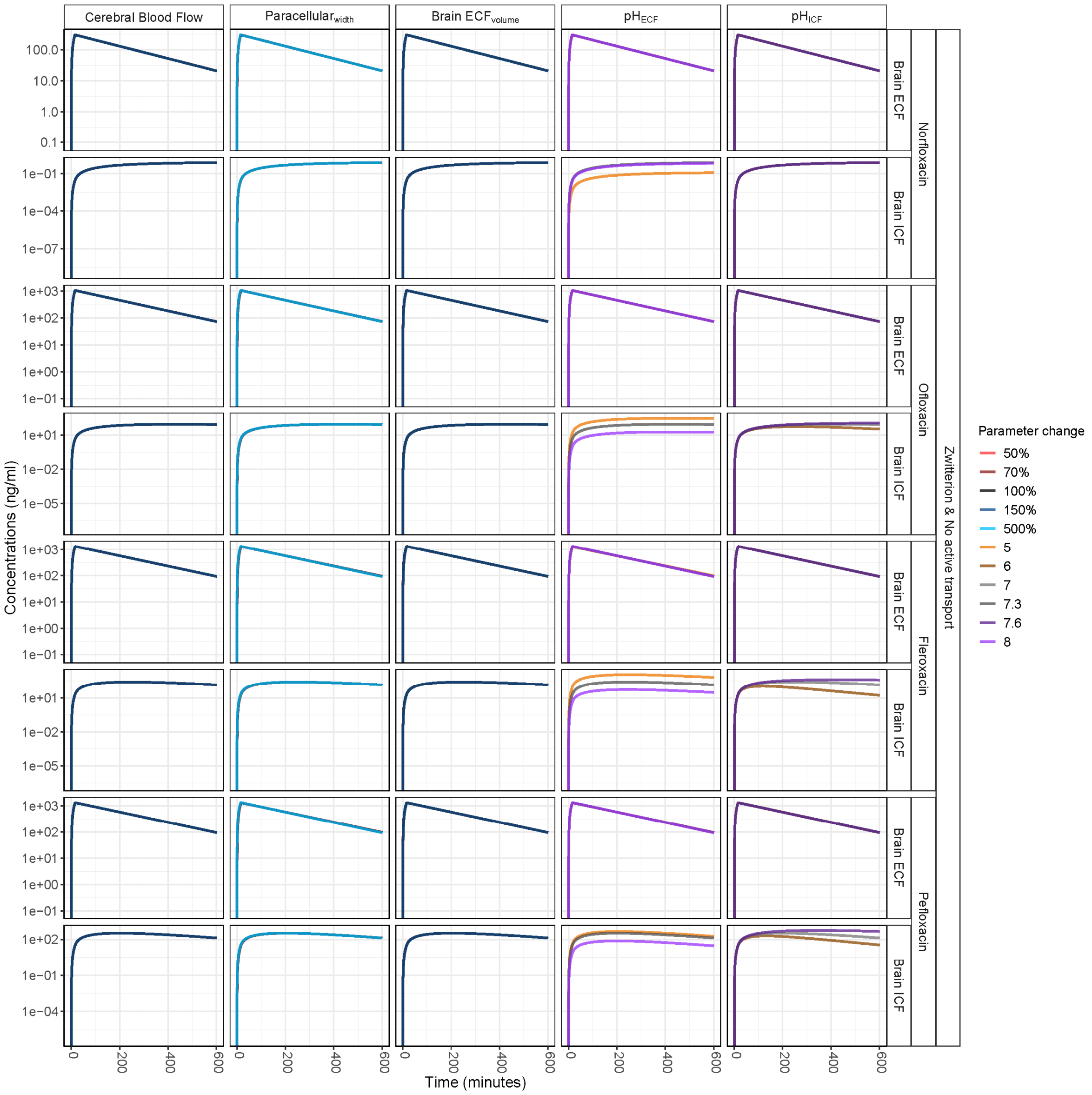

Figure S2. Continued

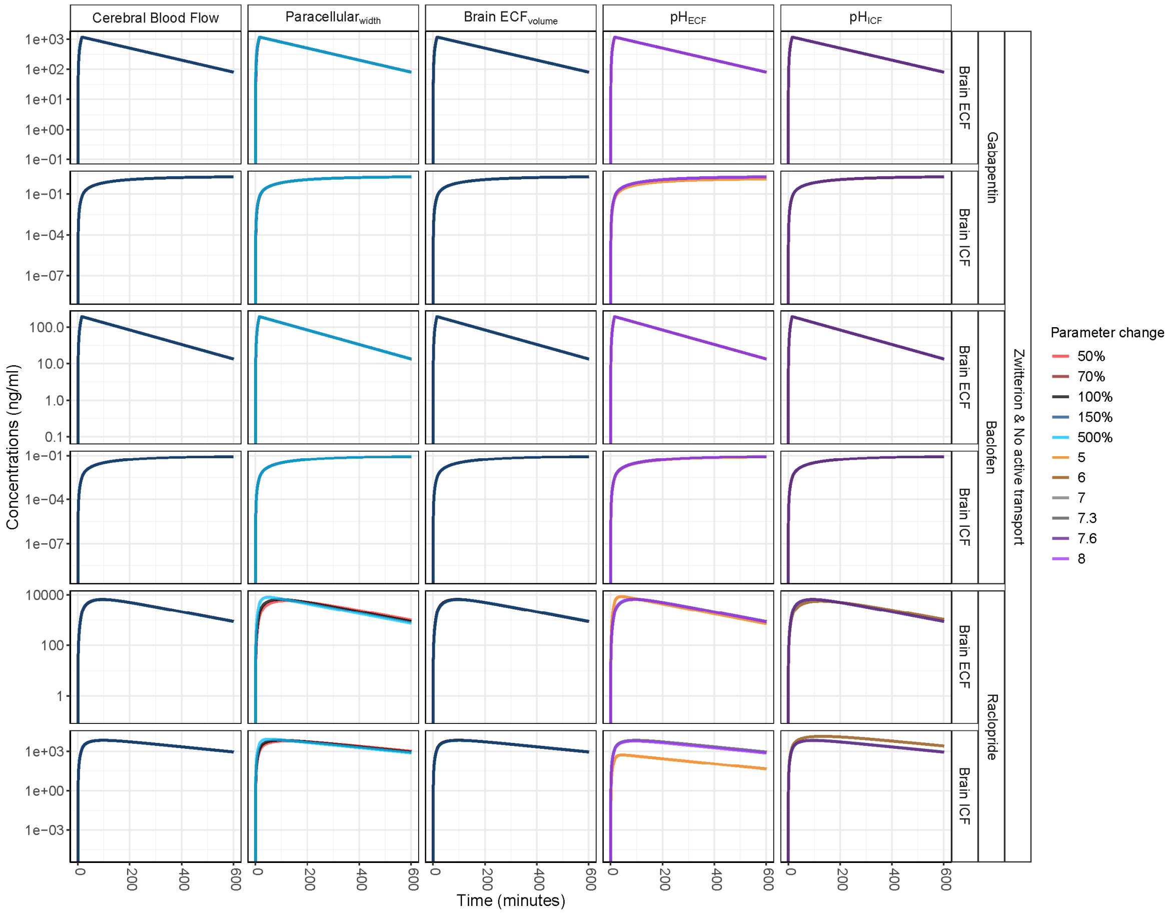

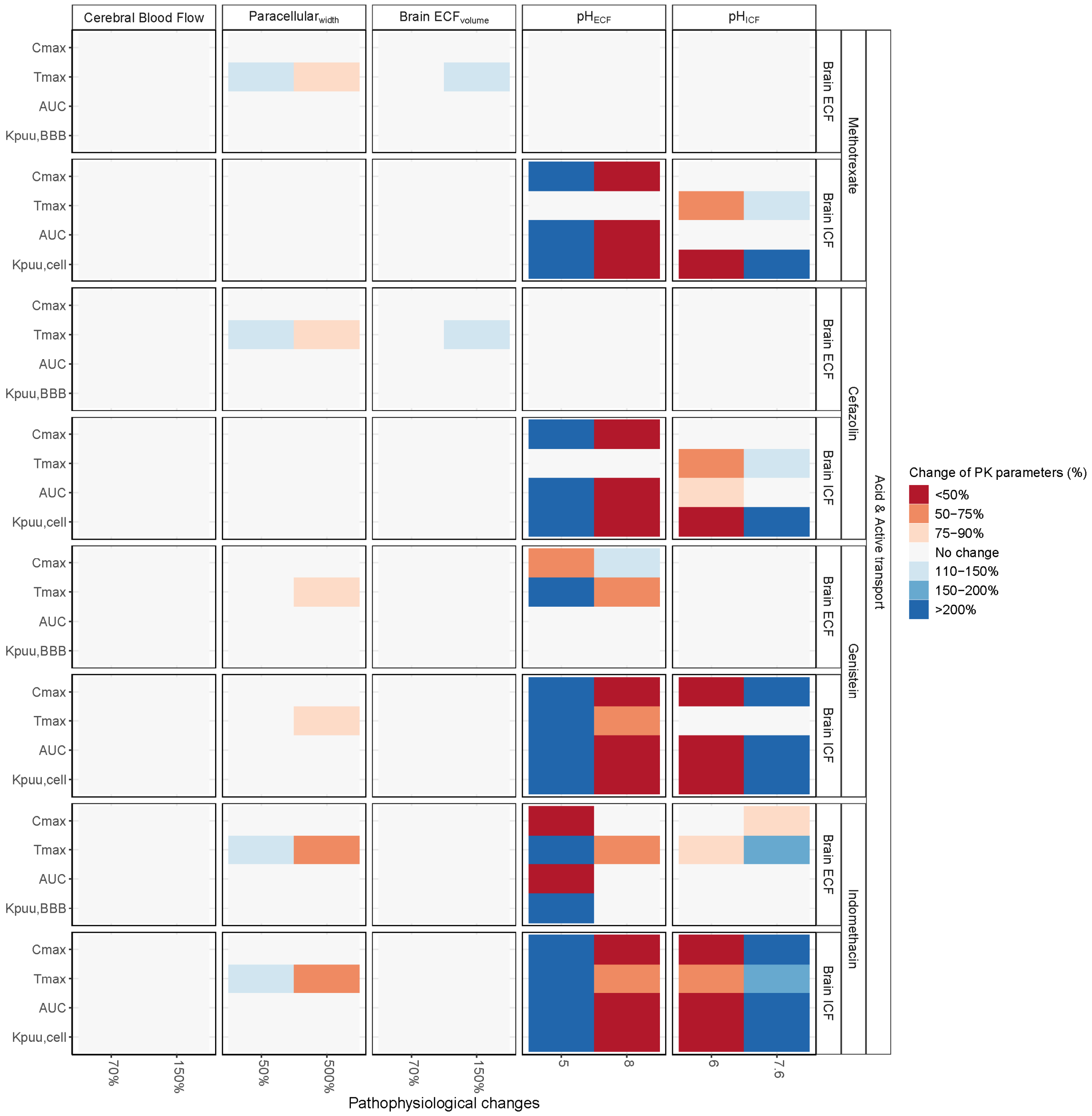

Figure S3. Heatmaps summarizing the effect of pathophysiological changes of CBF, $para_{radius}$ ($paracellular_{width}$), $brain_{ECF}$ volume, pH_{ECF}, and pH_{ICF} on brain pharmacokinetics parameters: C_{max}, T_{max}, AUC, $Kp_{uu,ECF}$, and $Kp_{uu,cell}$.

Figure S3. (Continued)

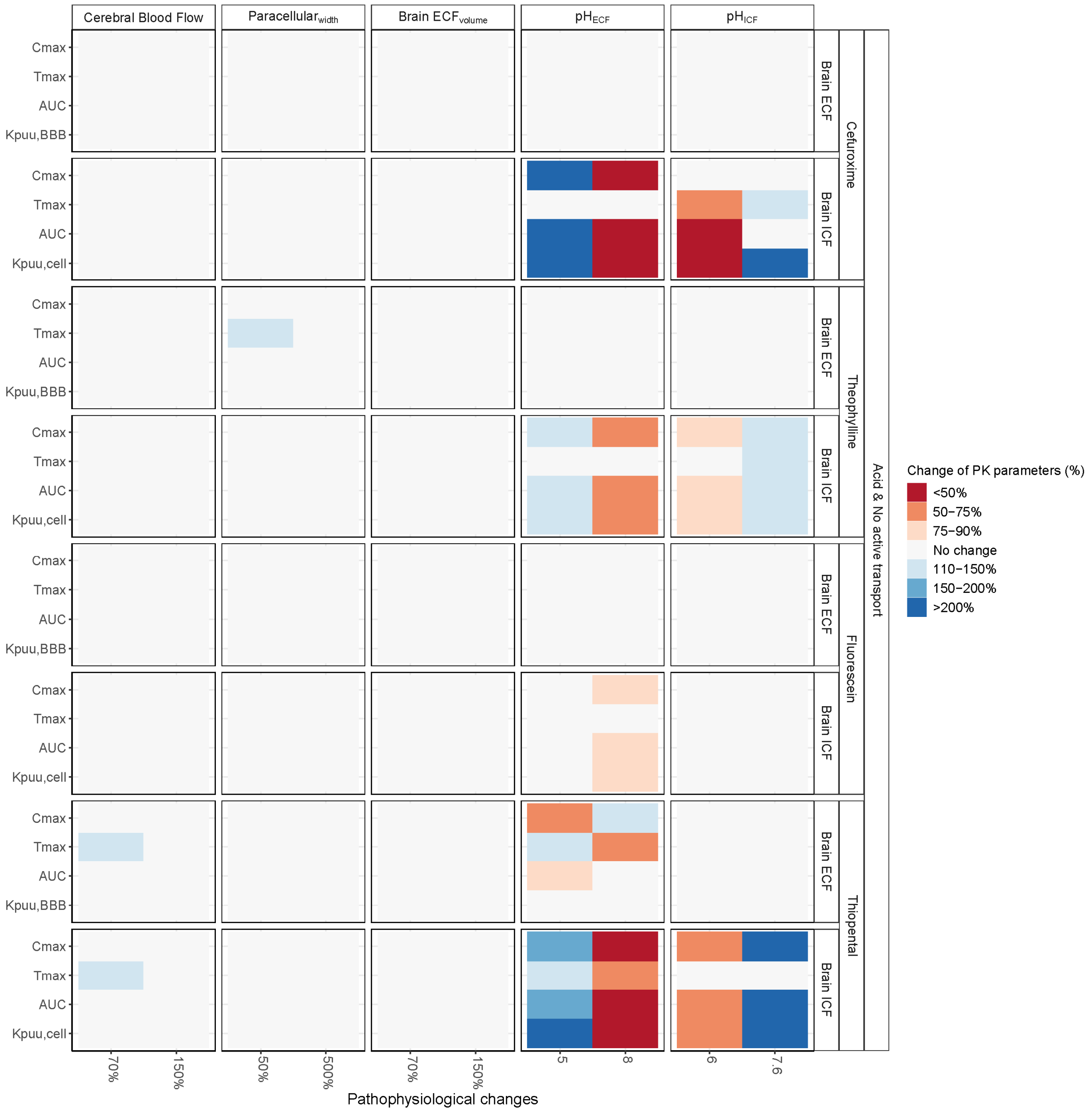

Figure S3. (Continued)

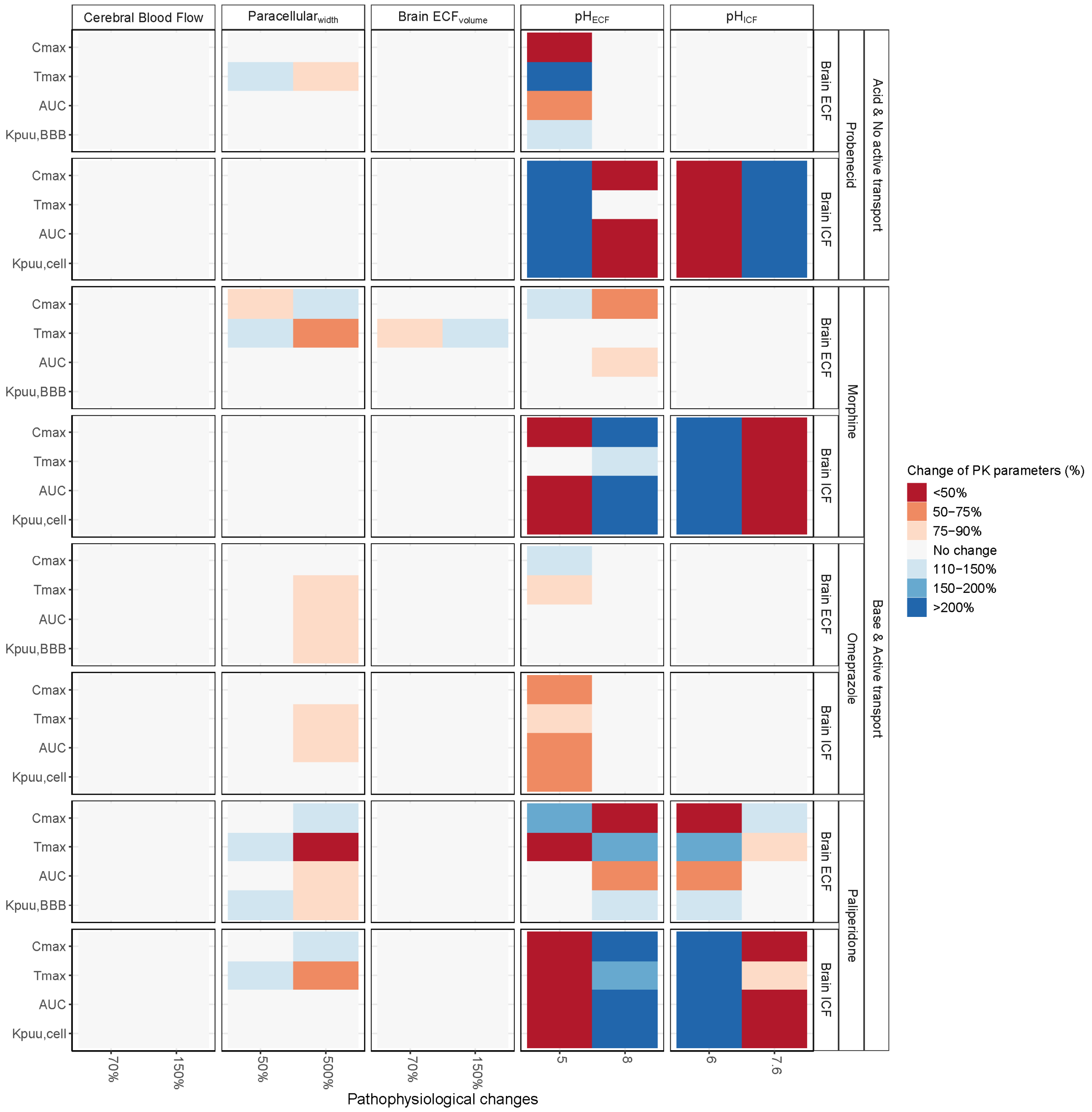

Figure S3. (Continued)

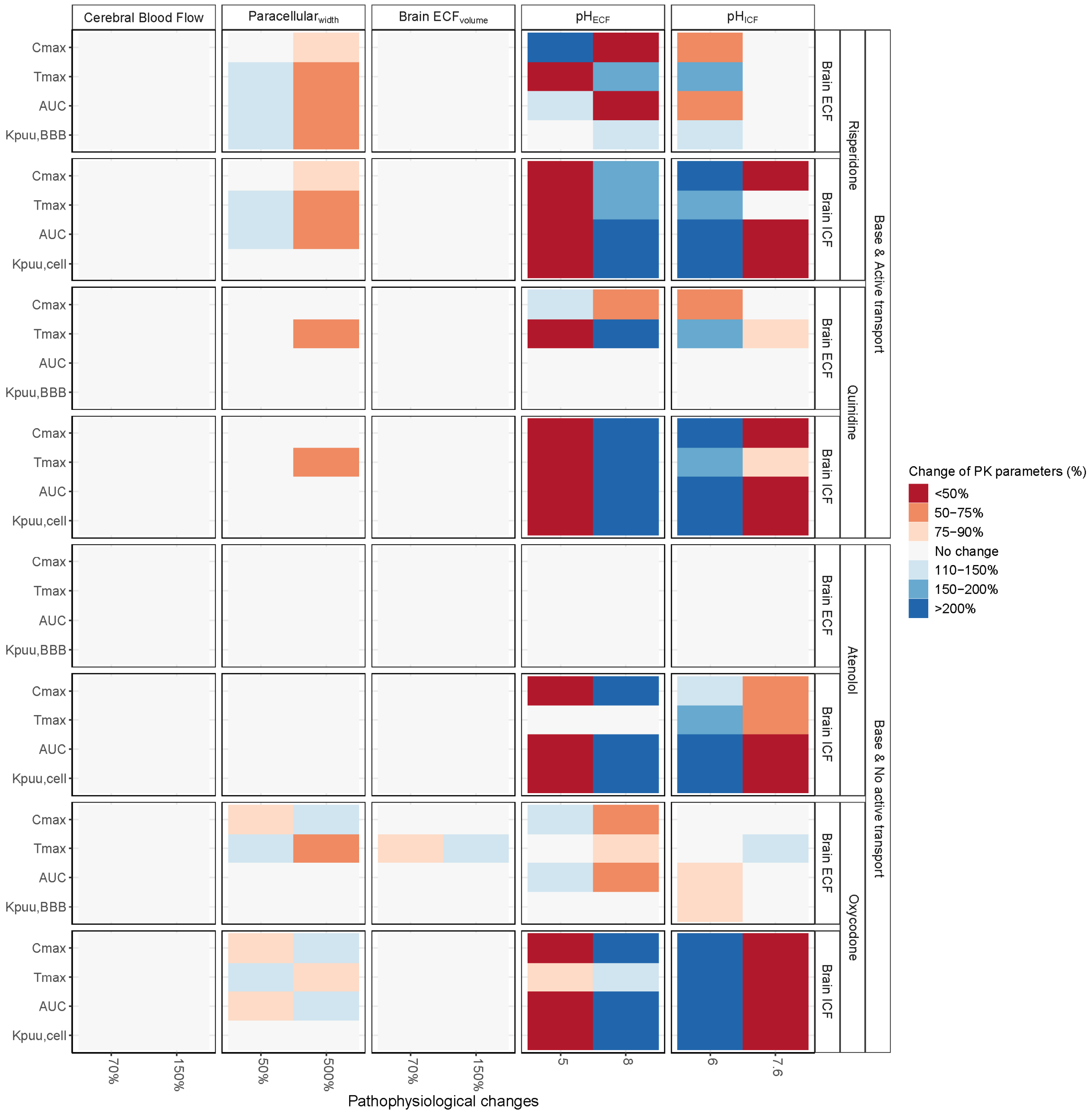

Figure S3. (Continued)

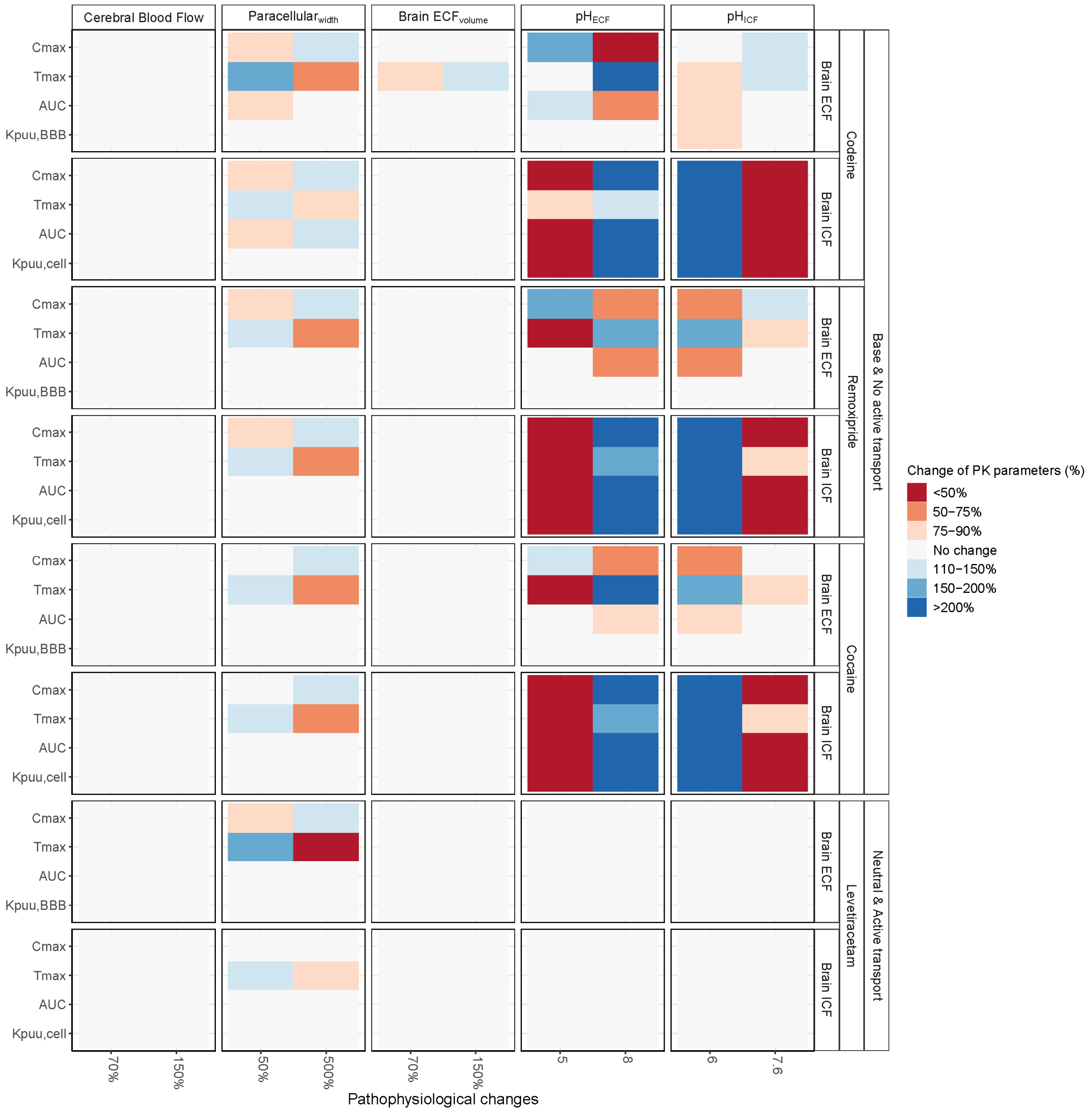

Figure S3. (Continued)

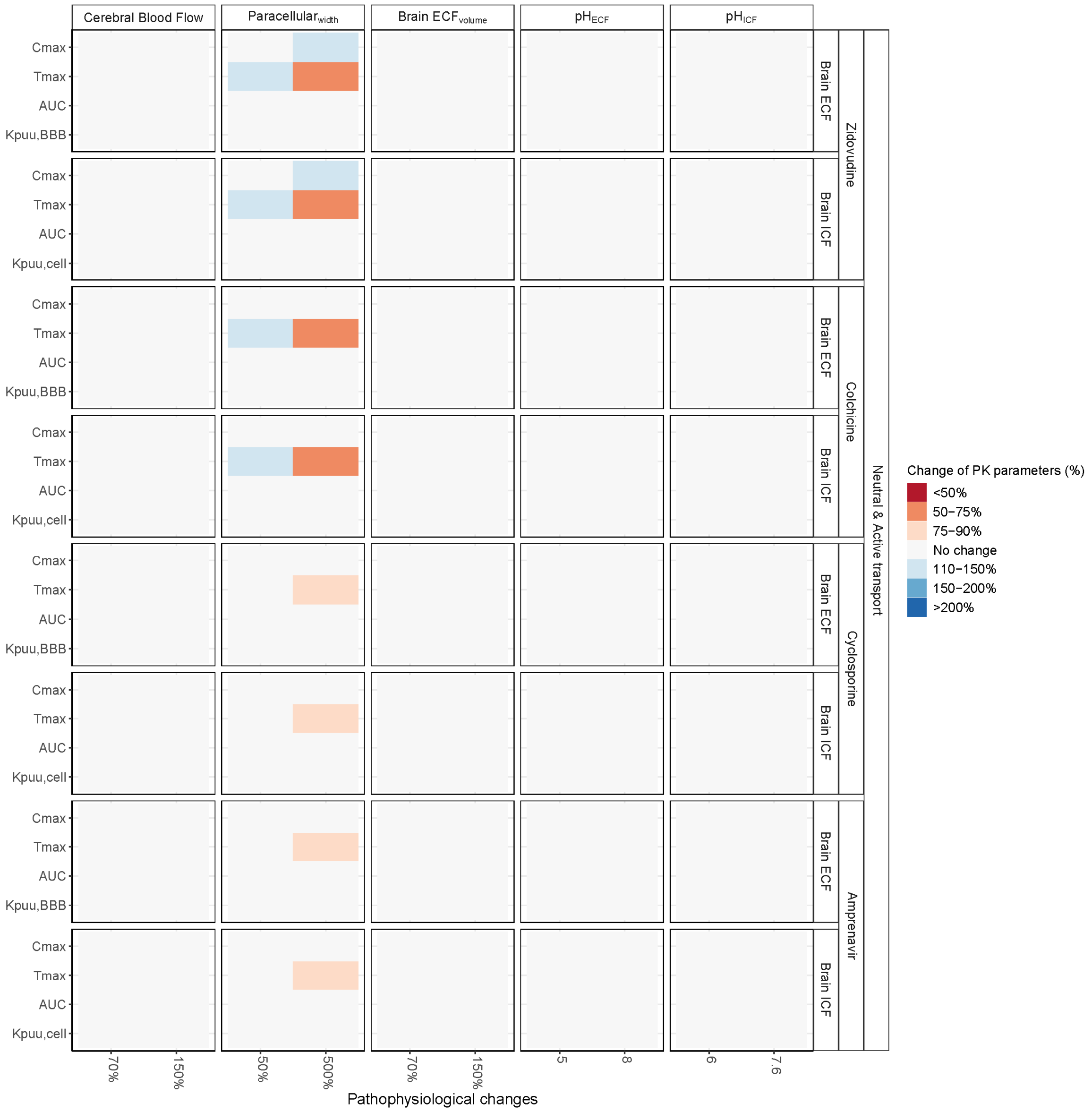

Figure S3. (Continued)

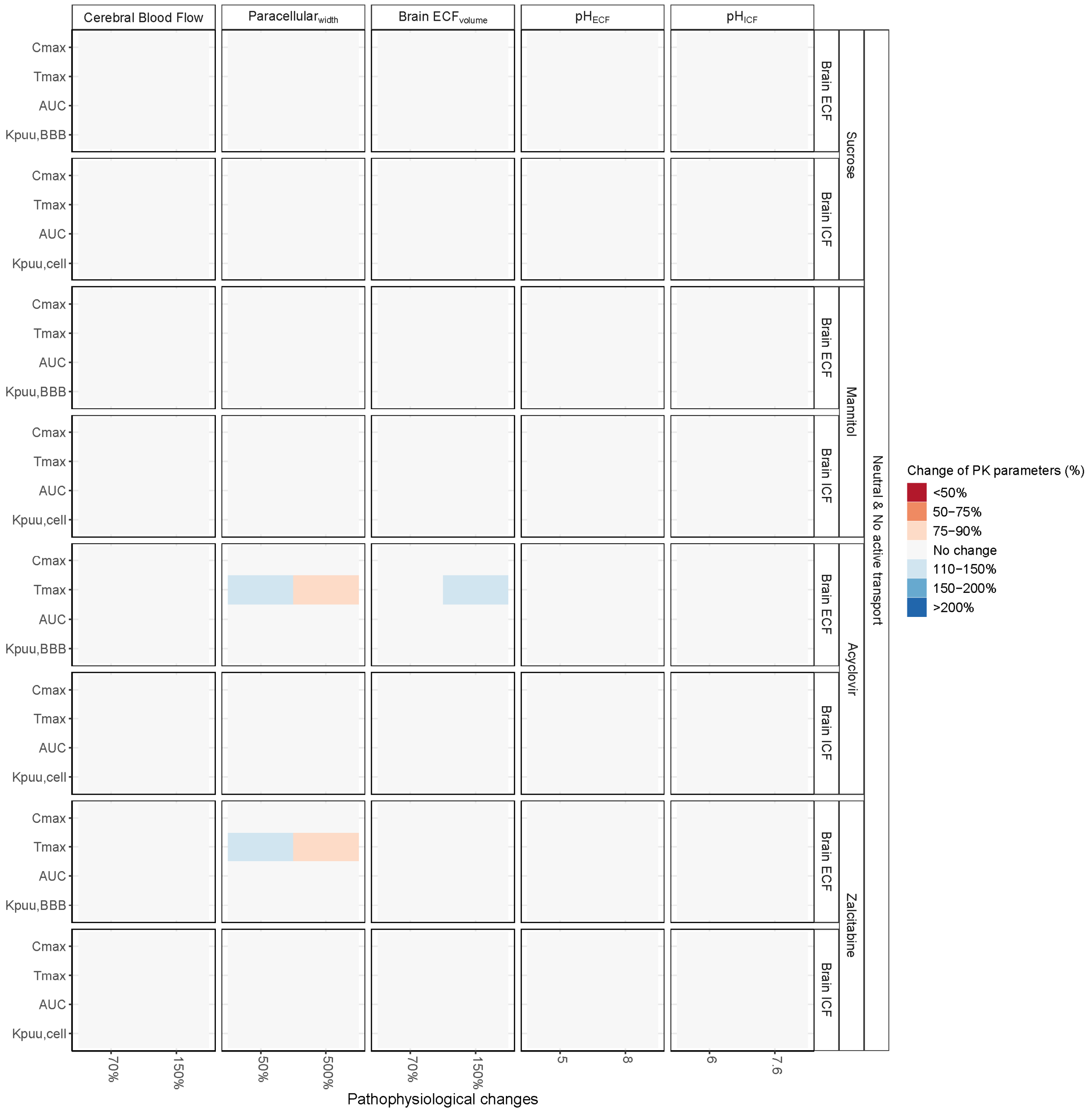

Figure S3. (Continued)

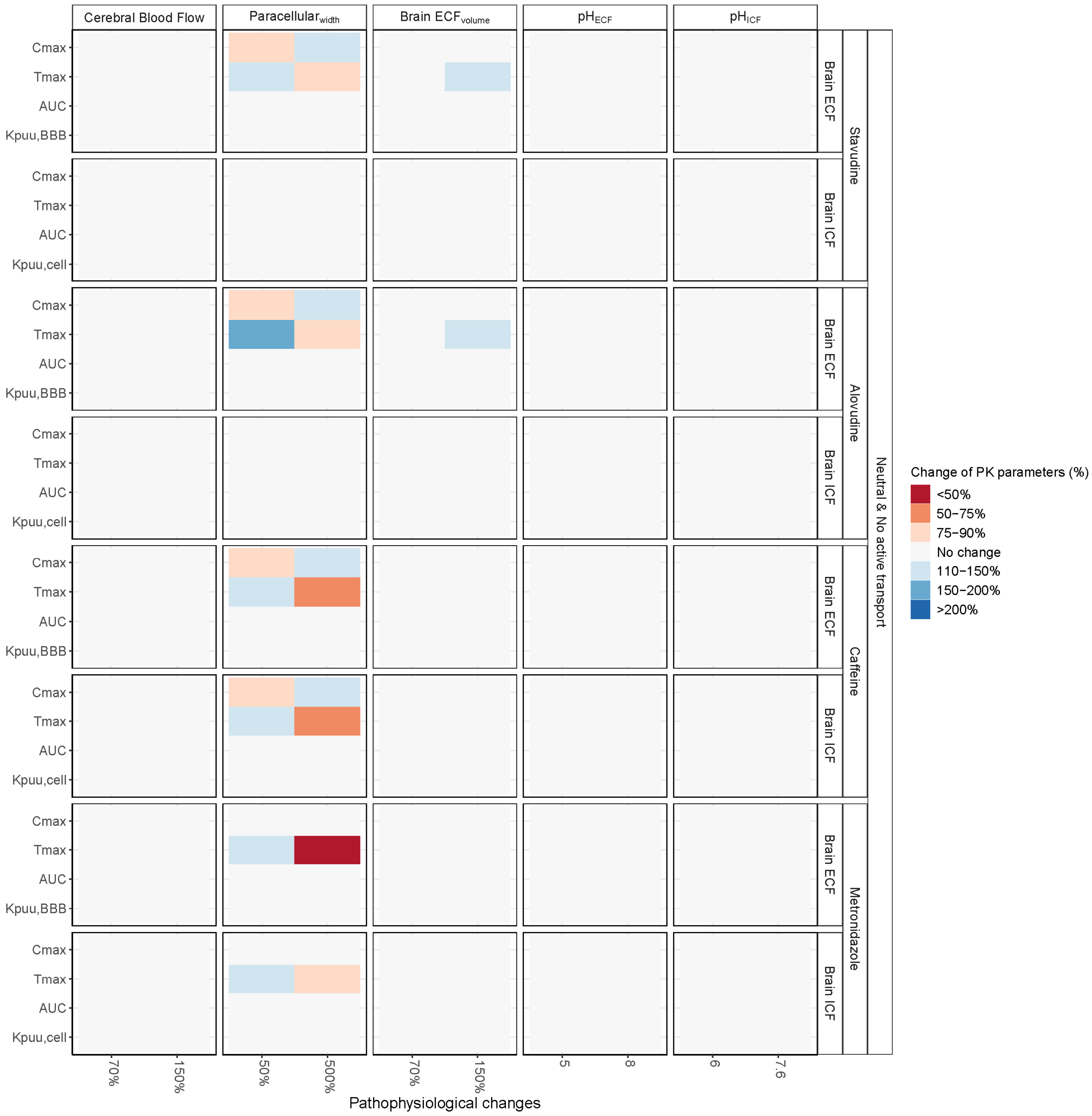

Figure S3. (Continued)

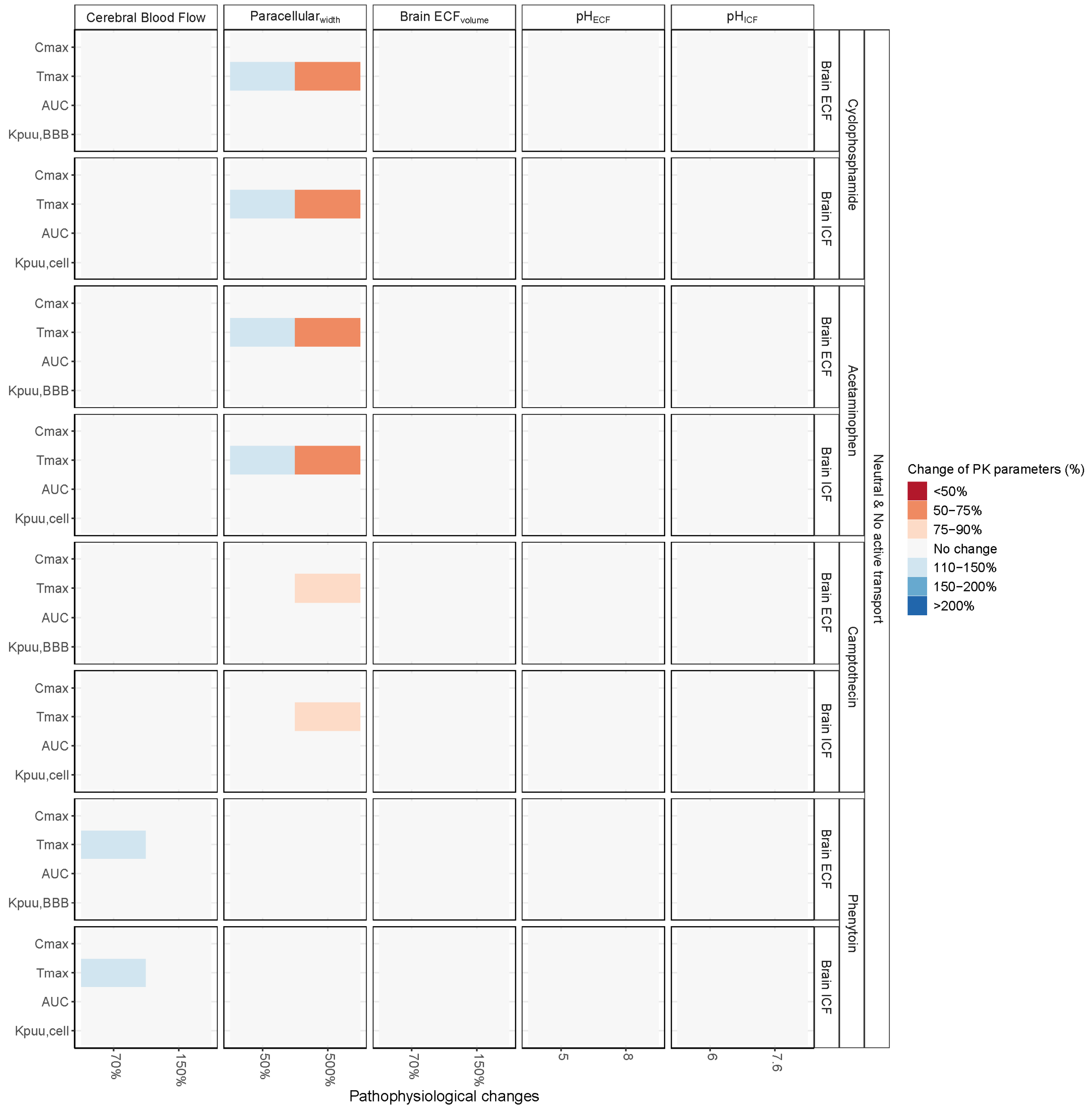

Figure S3. (Continued)

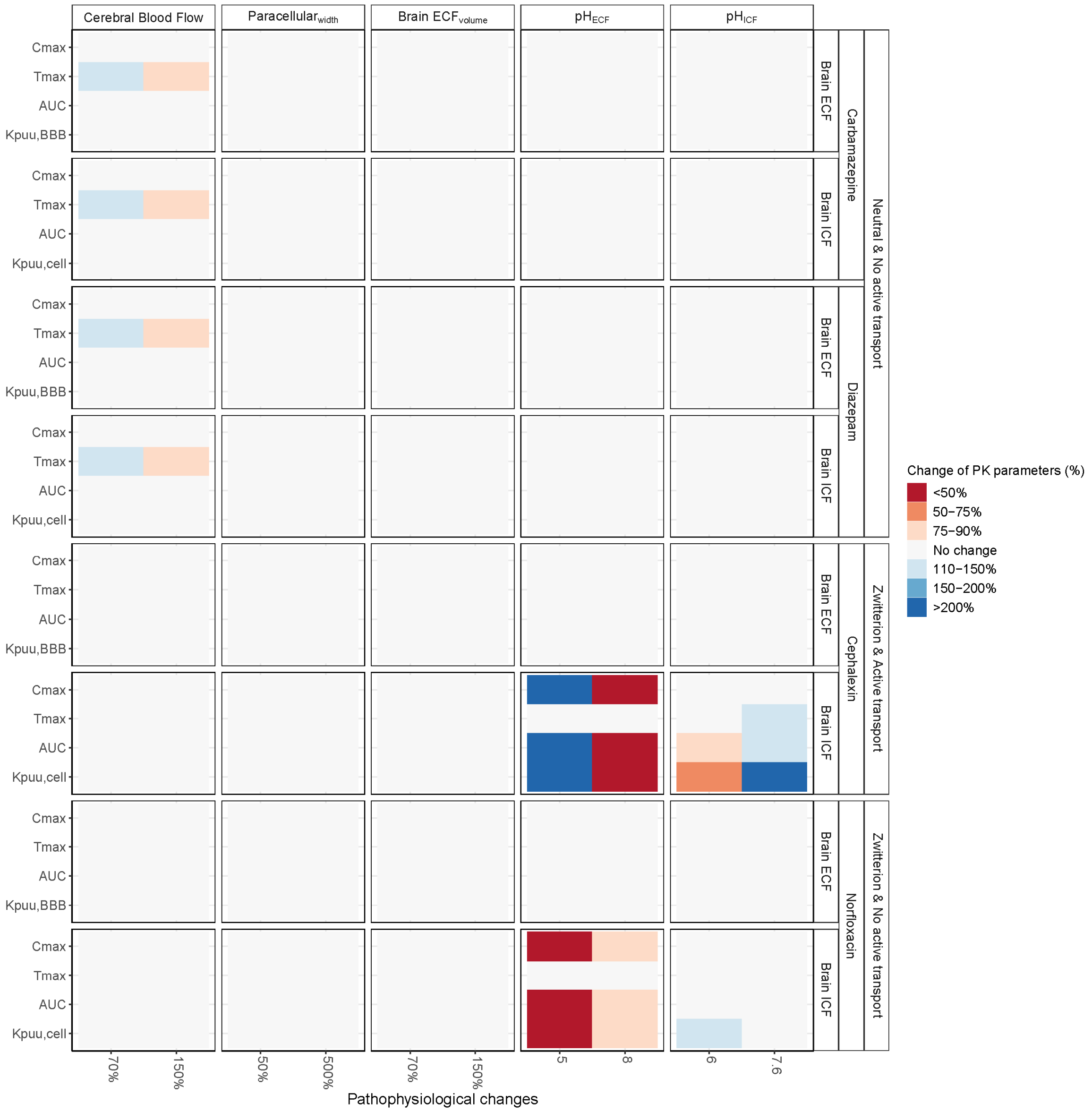

Figure S3. (Continued)

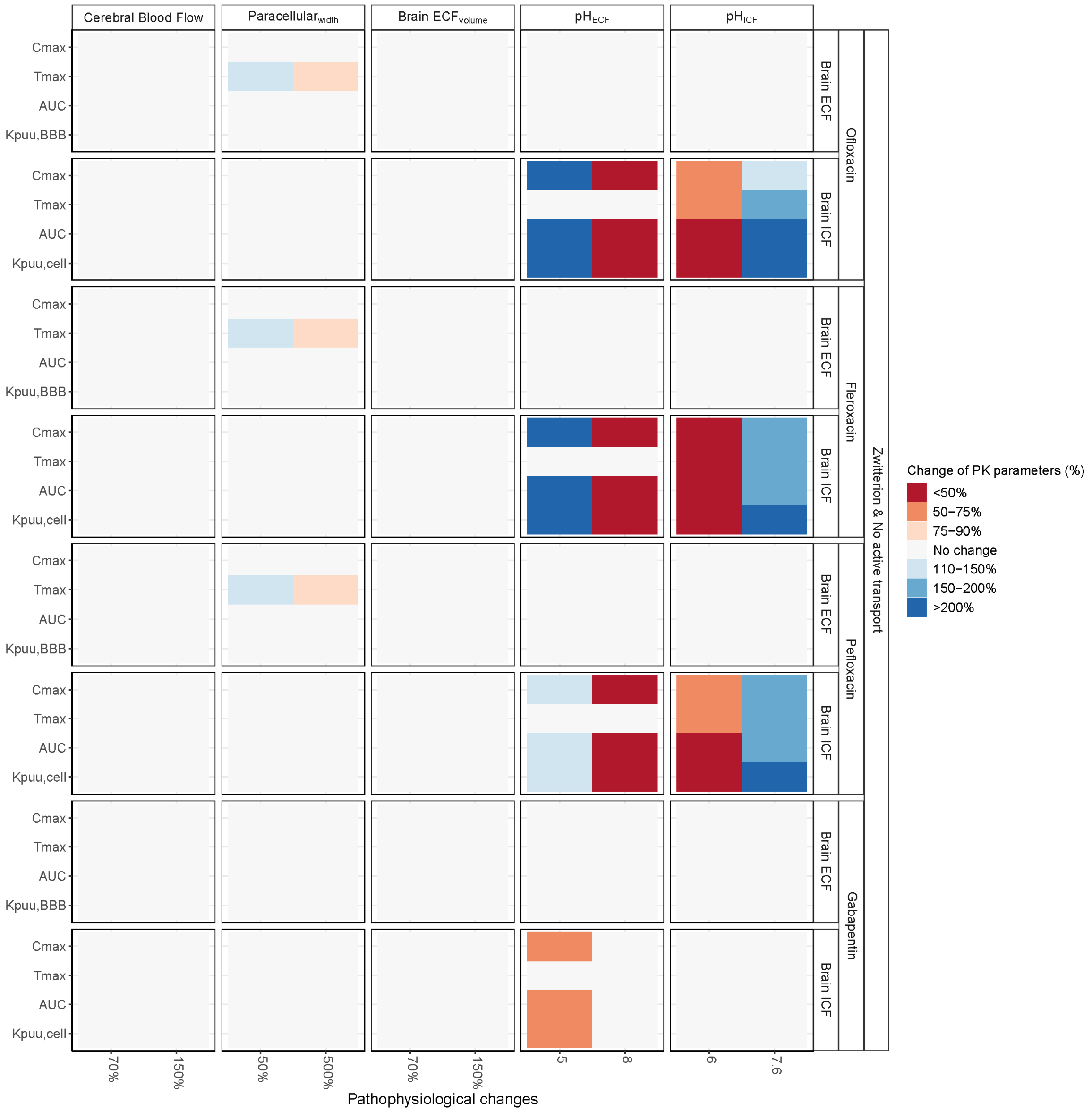

Figure S3. (Continued)

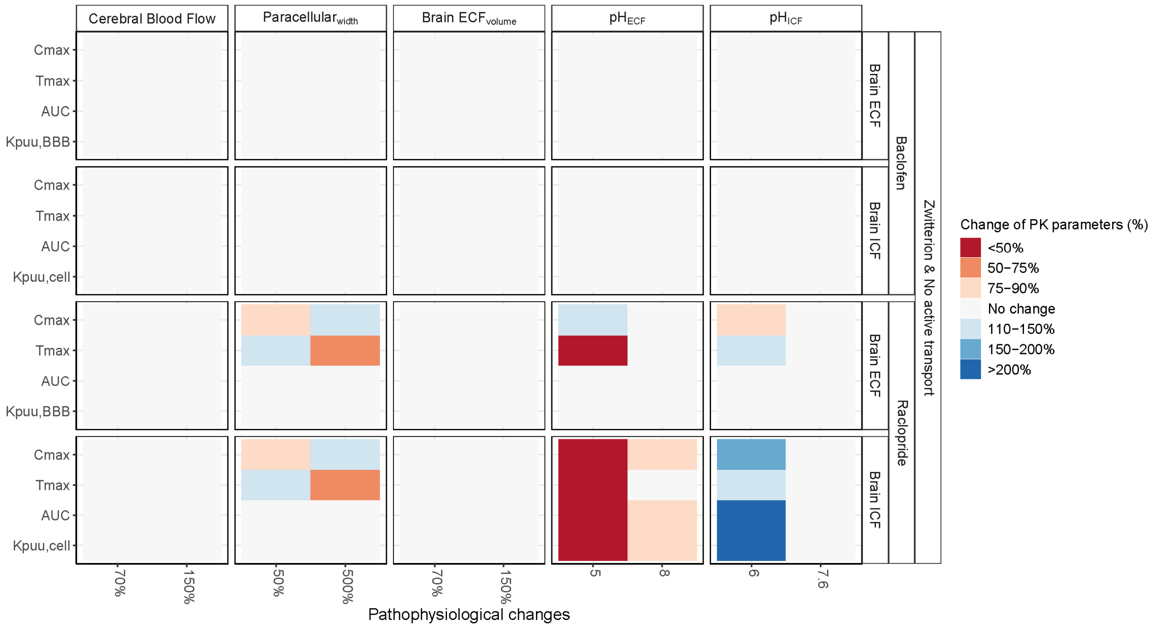

Table S1. Physicochemical properties, active transporter affinities, and BBB transport clearances of all 46 drugs.

Drug	Mwt	logP	Drug Ion Class	pk_a	pk_b	$Kp_{uu,ECF}$	$Kp_{uu,LV}$	$Kp_{uu,CM}$	BCRP	p-gp	OAT3	MRP4	CL_P	$CL_{T,ef}$	$CL_{T,in}$
Acetaminophen	151.2	0.91	Neutral	9.46	−4.4	0.51 [1]	0.51 [1]	0.51 [1]	-	-	-	-	54.93	91.03	19.64
Acyclovir	225.2	−1.76	Neutral	11.98	3.02	0.3 [2]	0.3 [2]	0.3 [2]	-	-	-	-	45.71	106.64	0.06
Alovudine	244.2	−0.6	Neutral	10.11	−3	0.29 [2]	0.29 [2]	0.29 [2]	-	-	-	-	44.04	110.19	0.76
Amprenavir	505.6	1.85	Neutral	13.61	2.39	0.076 [2]	0.076 [2]	0.076 [2]	-	X	-	-	31.49	517.39	151.18
Atenolol	266.3	0.16	Base	14.08	9.67	0.037 [1]	0.037 [1]	0.037 [1]	-	-	-	-	42.31	1101.56	0.02
Baclofen	213.7	1.3	Zwitterion	3.89	9.79	0.022 [2]	0.022 [2]	0.022 [2]	-	-	-	-	46.83	2081.76	<0.01
Caffeine	194.2	−0.07	Neutral	NA	−0.92	0.96 [2]	0.96 [2]	0.96 [2]	X	-	-	-	48.94	4.28	2.38
Camptothecin	348.4	1.74	Neutral	11.71	3.07	0.27 [2]	0.27 [2]	0.27 [2]	-	-	-	-	37.39	542.15	119.17
Carbamazepine	236.3	2.77	Neutral	15.96	−3.8	1.02 [2]	1.02 [2]	1.02 [2]	-	-	-	-	44.71	1105.03	1128.96
Cefazolin	454.5	−0.58	Acid	3.03	0.26	0.06 [2]	0.06 [2]	0.06 [2]	-	-	X	X	33.07	83.64	<0.01
Cefuroxime	424.4	−0.16	Acid	3.15	−1.1	0.042 [2]	0.042 [2]	0.042 [2]	-	-	-	-	34.13	778.38	<0.01
Cephalexin	347.4	0.65	Zwitterion	3.26	7.23	0.015 [2]	0.015 [2]	0.015 [2]	-	-	X	-	37.43	2735.53	<0.01
Cocaine	303.4	2.3	Base	NA	8.85	0.37 [2]	0.37 [2]	0.37 [2]	-	-	-	-	39.85	104.66	13.71
Codeine	299.4	1.39	Base	13.78	9.19	1 [2]	1 [2]	1 [2]	-	-	-	-	40.09	0.71	0.89
Colchicine	399.4	1.07	Neutral	15.06	−0.038	0.04 [2]	0.04 [2]	0.04 [2]	-	X	-	-	35.10	336.40	27.99
Cyclophosphamide	261.1	0.8	Neutral	12.78	−0.57	0.216 [3]	0.216 [3]	0.216 [3]	-	-	-	-	42.70	227.04	15.61
Cyclosporine	1202.6	1.4	Neutral	11.83	−2.4	0.023 [4]	0.023 [4]	0.023 [4]	-	X	-	-	21.12	743.86	57.14

Mwt: molecular weight (g/mol); logP: octanol-water partition coefficient; pK_a: acid dissociation coefficient; pK_b: base dissociation coefficient; $CL_{T,ef}$: transcellulr efflux clearance (in ml/min) at BBB; $CL_{T,in}$: transcellulr influx clearance (in ml/min) at BBB; CL_P: paracellular passive BBB clearance (in ml/min); X: active transporter substrate; p-gp: P-glycoprotein, MRP4: multi-drug-resistant protein-4, BCRP: breast cancer resistance protein, OAT3: organic anionic transporter 3. $CL_{T,ef}$, $CL_{T,in}$, and CL_P are calculated as described in [12,13].

[1] Saleh et al. Submitted. British Journal of Clinical Pharmacology. 2020. [6]

[2] Summerfield et al. The Journal of Pharmacology and Experimental Therapeutics. 2007. [7]

[3] Campagne et al. Journal of Pharmacy and Pharmaceutical Sciences. 2019. [8]

[4] Legg et al. Journal of Pharmacy and Pharmacology. 1987, Brophy et al. Journal of Neurotrauma. 2013, Zaghloul et al. Journal of Clinical Pharmacology. 1987. [9–11]

Drug	Mwt	logP	Drug Ion Class	pk_a	pk_b	$Kp_{uu,ECF}$	$Kp_{uu,LV}$	$Kp_{uu,CM}$	BCRP	p-gp	OAT3	MRP4	CL_p	$CL_{T,ef}$	$CL_{T,in}$
Diazepam	284.7	2.82	Neutral	NA	2.92	0.98 [2]	0.98 [2]	0.98 [2]	-	-	-	-	41.03	1256.12	1231.14
Fleroxacin	369.3	0.24	Zwitterion	5.44	6.06	0.15 [2]	0.15 [2]	0.15 [2]	-	-	-	-	36.39	206.32	0.05
Fluorescein	332.3	2.64	Acid	8.72	−3.7	0.018 [2]	0.018 [2]	0.018 [2]	-	-	-	-	38.21	46314.68	796.16
Gabapentin	171.2	1.25	Zwitterion	4.63	9.91	0.13 [2]	0.13 [2]	0.13 [2]	-	-	-	-	51.86	346.86	<0.01
Genistein	270.2	3.04	Acid	6.55	−5.3	0.04 [2]	0.04 [2]	0.04 [2]	X	X	-	-	42.03	1557.22	245.20
Indomethacin	357.8	4.27	Acid	3.79	−2.9	0.11 [1]	0.17 [1]	0.17 [1]	-	-	X	X	36.93	58.41	6.95
Levetiracetam	170.2	−0.64	Neutral	16.09	−1.6	0.31 [2]	0.31 [2]	0.31 [2]	-	X	-	X	52.01	3.73	0.69
Mannitol	182.2	−3.1	Neutral	12.59	−3	0.014 [2]	0.014 [2]	0.014 [2]	-	-	-	-	50.41	3549.99	<0.01
Methotrexate	454.4	−1.85	Acid	3.41	2.81	0.018 [1]	0.0066 [1]	0.0024 [1]	X	X	X	X	33.08	63.57	<0.01
Metronidazole	171.2	−0.02	Neutral	15.44	3.09	0.23 [2]	0.23 [2]	0.23 [2]	-	-	-	-	51.88	184.96	2.65
Morphine	285.3	0.87	Base	10.26	9.12	0.23 [1]	0.23 [1]	0.23 [1]	-	X	-	-	40.99	30.21	0.34
Norfloxacin	319.3	−1.03	Zwitterion	5.77	8. 68	0.034 [2]	0.034 [2]	0.034 [2]	-	-	-	-	38.92	1105.46	<0.01
Ofloxacin	361.4	−0.39	Zwitterion	5.45	6.2	0.12 [2]	0.12 [2]	0.12 [2]	-	-	-	-	36.76	269.45	0.01
Omeprazole	345.4	2.23	Base	9.29	4.77	0.15 [2]	0.15 [2]	0.15 [2]	X	X	-	-	37.53	538.80	338.64
Oxycodone	315.4	0.7	Base	13.57	8.77	1.03 [1]	0.65 [1]	0.65 [1]	-	-	-	-	39.14	0.41	1.82
Paliperidone	426.5	2.3	Base	13.74	8.76	0.5 [1]	0.5 [1]	0.5 [1]	-	X	-	-	34.06	14.68	16.73
Pefloxacin	333.4	0.27	Zwitterion	5.66	6.47	0.15 [2]	0.15 [2]	0.15 [2]	-	-	-	-	38.15	216.50	0.08
Phenytoin	252.3	2.47	Neutral	9.47	−9	1 [1]	1 [1]	1 [1]	-	-	-	-	43.38	573.78	572.79
Probenecid	285.4	3.21	Acid	3.53	NA	0.2 [2]	0.2 [2]	0.2 [2]	-	-	-	-	40.99	165.65	0.39
Quinidine	324.4	3.44	Base	13.89	9.05	0.0674 [5]	0.0678 [5]	0.0678 [5]	-	X	X	-	38.63	500.77	103.01
Raclopride	347.2	3.19	Zwitterion	6.26	8.47	1.1 [1]	1.1 [1]	1.1 [1]	-	-	-	-	37.44	14.50	19.96
Remoxipride	371.3	2.1	Base	13.06	8.4	0.8 [1]	0.8 [1]	0.8 [1]	-	-	-	-	36.30	38.33	23.60
Risperidone	410.5	3.27	Base	NA	8.76	0.97 [1]	0.97 [1]	0.97 [1]	-	X	-	-	34.66	30.81	136.24

[5] Nagaya et al. Drug Metabolism and Pharmacokinetic. 2016. [12]

Drug	Mwt	logP	Drug Ion Class	pk_a	pk_b	$Kp_{uu,ECF}$	$Kp_{uu,LV}$	$Kp_{uu,CM}$	BCRP	p-gp	OAT3	MRP4	CL_p	$CL_{T,ef}$	$CL_{T,in}$
Stavudine	224.2	−0.72	Neutral	9.95	−3	0.33 [2]	0.33 [2]	0.33 [2]	-	-	-	-	45.80	94.53	0.58
Sucrose	342.3	−3.7	Neutral	11.84	−3	0.0027 [2]	0.0027 [2]	0.0027 [2]	-	-	-	-	37.69	13921.70	<0.01
Theophylline	180.2	−0.02	Acid	7.82	−0.78	0.05 [2]	0.05 [2]	0.05 [2]	-	-	-	-	50.66	1000.80	1.92
Thiopental	242.3	2.85	Acid	7.2	−3	0.9 [2]	0.9 [2]	0.9 [2]	-	-	-	-	44.19	569.06	508.22
Zalcitabine	211.2	−1.3	Neutral	14.67	0.18	0.19 [2]	0.19 [2]	0.19 [2]	-	-	-	-	47.08	201.37	0.17
Zidovudine	267.2	0.05	Neutral	9.96	−3	0.15 [2]	0.15 [2]	0.15 [2]	X	X	X	X	42.24	9.15	3.08

Table S2. Mean protein expression levels [1] (in fmol/μg total protein) of relevant transporters at the BBB.

	Human	References	Rat	References	Human:rat
p-gp	4.21	[1–3]	19.28	[4,5]	0.22
MRP4	0.25	[2,3]	1.74	[4,5]	0.15
BCRP	5.50	[1–3]	4.95	[5]	1.11
OAT3	0.27	[1]	2.13	[5]	0.13

[1] Experimentally-measured Kp_{uu} values from rats were used to account for active transport at the blood-brain barrier. These were translated to predict human BBB active transport using the difference in expression between rats and humans of the four main transporters (p-gp, BCRP, MRP4, OAT3) at the BBB. Information on drug affinities to the four transporters were available from Drugbank database and were manually checked. This translation procedure is described in more details in [13]. Transporters functionality were assumed the same between rats and humans.

References

1. Al-Majdoub, Z.M.; Al Feteisi, H.; Achour, B.; Warwood, S.; Neuhoff, S.; Rostami-Hodjegan, A.; Barber, J. Proteomic Quantification of Human Blood–Brain Barrier SLC and ABC Transporters in Healthy Individuals and Dementia Patients. *Mol. Pharm.* **2019**, doi:10.1021/acs.molpharmaceut.8b01189.
2. Shawahna, R.; Uchida, Y.; Declèves, X.; Ohtsuki, S.; Yousif, S.; Dauchy, S.; Jacob, A.; Chassoux, F.; Daumas-Duport, C.; Couraud, P.O.; et al. Transcriptomic and quantitative proteomic analysis of transporters and drug metabolizing enzymes in freshly isolated human brain microvessels. *Mol. Pharm.* **2011**, *8*, 1332–1341, doi:10.1021/mp200129p.
3. Uchida, Y.; Ohtsuki, S.; Katsukura, Y.; Ikeda, C.; Suzuki, T.; Kamiie, J.; Terasaki, T. Quantitative targeted absolute proteomics of human blood-brain barrier transporters and receptors. *J. Neurochem.* **2011**, *117*, 333–345, doi:10.1111/j.1471-4159.2011.07208.x.
4. Al Feteisi, H.; Al-Majdoub, Z.M.; Achour, B.; Couto, N.; Rostami-Hodjegan, A.; Barber, J. Identification and quantification of blood–brain barrier transporters in isolated rat brain microvessels. *J. Neurochem.* **2018**, *146*, 670–685, doi:10.1111/jnc.14446.
5. Hoshi, Y.; Uchida, Y.; Tachikawa, M.; Inoue, T.; Ohtsuki, S.; Terasaki, T. Quantitative Atlas of Blood–Brain Barrier Transporters, Receptors, and Tight Junction Proteins in Rats and Common Marmoset. *J. Pharm. Sci.* **2013**, *102*, 3343–3355, doi:10.1002/jps.
6. Saleh, M.A.A.; Loo, C.F.; Elassaiss-Schaap, J.; De Lange, E.C.M. Lumbar cerebrospinal fluid-to-Brain extracellular fluid surrogacy is context-specific: insights from LeiCNS-PK3.0 simulations. Under review. *Br. J. Clin. Pharmacol.* **2020**.
7. Summerfield, S.G.; Read, K.; Begley, D.J.; Obradovic, T.; Hidalgo, I.J.; Coggon, S.; Lewis, A. V.; Porter, R.A.; Jeffrey, P. Central Nervous System Drug Disposition: The Relationship between in Situ Brain Permeability and Brain Free Fraction. *J. Pharmacol. Exp. Ther.* **2007**, *322*, 205–213, doi:10.1124/jpet.107.121525.
8. Campagne, O.; Davis, A.; Zhong, B.; Nair, S.; Haberman, V.; Patel, Y.T.; Janke, L.; Roussel, M.F.; Stewart, C.F. CNS Penetration of Cyclophosphamide and Metabolites in Mice Bearing Group 3 Medulloblastoma and Non-tumor Bearing Mice. *J. Pharm. Pharm. Sci.* **2019**, *22*, 612–629, doi:10.18433/JPPS30608.
9. Legg, B.; Rowland, M. Cyclosporin: measurement of fraction unbound in plasma. *J. Pharm. Pharmacol.* **1987**, *39*, 599–603, doi:10.1111/j.2042-7158.1987.tb03436.x.
10. Brophy, G.M.; Mazzeo, A.T.; Brar, S.; Alves, O.L.; Bunnell, K.; Gilman, C.; Karnes, T.; Hayes, R.L.; Bullock, R. Exposure of Cyclosporin A in Whole Blood , Cerebral Spinal Fluid , and Brain Extracellular Fluid Dialysate in Adults with Traumatic Brain Injury. *J. Neurotrauma* **2013**, *30*, 1484–1489, doi:10.1089/neu.2012.2524.
11. Zaghloul, I.; Ptachcinski, R.J.; Burckart, G.J.; Van Thiel, D.; Starzel Th., E.; Venkataramanan, R. Blood protein binding of cyclosporine in transplant patients. *J. Clin. Pharmacol.* **1987**, *27*, 240–242, doi:10.1002/j.1552-4604.1987.tb02192.x.
12. Nagaya, Y.; Nozaki, Y.; Takenaka, O.; Watari, R.; Kusano, K.; Yoshimura, T.; Kusuhara, H. Investigation of utility of cerebrospinal fluid drug concentration as a surrogate for interstitial fluid concentration using microdialysis coupled with cisternal cerebrospinal fluid sampling in wild-type and Mdr1a(-/-) rats. *Drug Metab. Pharmacokinet.* **2016**, *31*, 57–66, doi:10.1016/j.dmpk.2015.10.003.
13. Yamamoto, Y.; Välitalo, P.A.; Wong, Y.C.; Huntjens, D.R.; Proost, J.H.; Vermeulen, A.; Krauwinkel, W.; Beukers, M.W.; Kokki, H.; Kokki, M.; et al. Prediction of human CNS pharmacokinetics using a physiologically-based pharmacokinetic modeling approach. *Eur. J. Pharm. Sci.* **2018**, *112*, 168–179, doi:10.1016/j.ejps.2017.11.011.
